# CIRI‐Deep Enables Single‐Cell and Spatial Transcriptomic Analysis of Circular RNAs with Deep Learning

**DOI:** 10.1002/advs.202308115

**Published:** 2024-02-02

**Authors:** Zihan Zhou, Jinyang Zhang, Xin Zheng, Zhicheng Pan, Fangqing Zhao, Yuan Gao

**Affiliations:** ^1^ National Genomics Data Center & CAS Key Laboratory of Genome Sciences and Information Beijing Institute of Genomics Chinese Academy of Sciences and China National Center for Bioinformation Beijing 100101 China; ^2^ Beijing Institutes of Life Science Chinese Academy of Sciences Beijing 100101 China; ^3^ University of Chinese Academy of Sciences Beijing 100101 China; ^4^ Center for Computational Biology Flatiron Institute New York 10010 USA

**Keywords:** circular RNA, deep learning, single cell RNA‐seq, spatial transcriptome, splicing

## Abstract

Circular RNAs (circRNAs) are a crucial yet relatively unexplored class of transcripts known for their tissue‐ and cell‐type‐specific expression patterns. Despite the advances in single‐cell and spatial transcriptomics, these technologies face difficulties in effectively profiling circRNAs due to inherent limitations in circRNA sequencing efficiency. To address this gap, a deep learning model, CIRI‐deep, is presented for comprehensive prediction of circRNA regulation on diverse types of RNA‐seq data. CIRI‐deep is trained on an extensive dataset of 25 million high‐confidence circRNA regulation events and achieved high performances on both test and leave‐out data, ensuring its accuracy in inferring differential events from RNA‐seq data. It is demonstrated that CIRI‐deep and its adapted version enable various circRNA analyses, including cluster‐ or region‐specific circRNA detection, BSJ ratio map visualization, and trans and cis feature importance evaluation. Collectively, CIRI‐deep's adaptability extends to all major types of RNA‐seq datasets including single‐cell and spatial transcriptomic data, which will undoubtedly broaden the horizons of circRNA research.

## Graphical abstract

Circular RNAs (circRNAs) are an important class of transcripts that are difficult to detect in single‐cell or spatial transcriptomic data. This study develops a deep learning model CIRI‐deep to predict differentially spliced circRNAs on diverse types of RNA‐seq data. CIRI‐deep enables circRNA analysis including cluster‐ or region‐specific circRNA detection, BSJ ratio map visualization, and trans and cis feature importance evaluation.

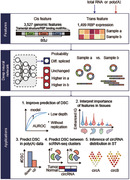

## 1 Introduction

Circular RNAs (circRNAs) are a type of RNA molecules generated through the covalent ligation of a downstream 5′ splice site (donor site) and an upstream 3′ splice site (acceptor site), a process known as back‐splicing.^[^ ^1^ ^]^ CircRNAs are widely expressed in high diversity across various species and tissues and have been proved to function as microRNA decoys,^[^ ^1^, ^2^ ^]^ protein scaffolds and protein encoders.^[^ ^3^ ^]^ As critical modulators of various biological processes, circRNAs have been reported to be precisely regulated and involved in cell cycle progression, epithelial‐mesenchymal transition, tissue development and tumor progression.^[^ ^3^, ^4^ ^]^ The production of circRNAs occurs through the spliceosome‐mediated process, typically requiring canonical splicing signals for exon circularization.^[^ ^5^ ^]^ The biogenesis of circRNA is regulated during splicing and involves both cis elements and trans factors. For example, studies have shown that Alu or complementary sequences in flanking introns promote back‐splicing,^[^ ^4^, ^6^ ^]^ and RNA binding proteins (RBPs), such as *QKI* and *HNRNPL*,^[^ ^4^, ^7^ ^]^ also affect circRNA biogenesis by binding to flanking introns and bridging distal splicing sites. Meanwhile, back‐splicing competes with and interacts with canonical splicing. Enhanced distal splicing signals promote exon inclusion in linear transcripts, consequently impeding circRNA biogenesis,^[^ ^8^ ^]^ whereas several instances of back‐splicing events have been reported to facilitate exon skipping.^[^ ^9^ ^]^

Similar to the commonly used percent spliced in (*PSI*) value for alternative splicing, the ratio of back‐splicing junction (BSJ) reads to all related reads at the junction is calculated in circRNA analysis.^[^ ^10^ ^]^ This junction ratio serves as a quantitative measure to assess the regulatory relationship and splicing preference between back‐splicing and canonical splicing. Accurate detection and quantification of circRNAs and their junction ratio rely on the identification of BSJ reads.^[^ ^11^ ^]^ However, circRNAs are often expressed at relatively low abundance compared to their linear counterparts,^[^ ^12^ ^]^ and existing circRNA analysis tools vary widely in sensitivity for different types of RNA‐seq data and are typically insensitive for samples with limited sequencing depth.^[^ ^13^ ^]^ For example, circRNAs are largely depleted by the prevalent poly(A) selection step in cDNA library construction due to the lack of polyadenylated tails, which hinders circRNA detection in most publicly available RNA‐seq datasets. Recent advancements in single‐cell RNA‐seq (scRNA‐seq) technology have revolutionized the study of cellular heterogeneity, cell lineage commitment, and the tumor microenvironment.^[^ ^14^ ^]^ Several studies have revealed the tissue‐ and development‐stage‐specific expression of circRNAs,^[^ ^2^, ^15^ ^]^ while limited studies have explored circRNA heterogeneity at the single‐cell level.^[^ ^16^ ^]^ This limitation arises primarily from the inability to detect and quantify circRNAs in low‐depth and poly(A) selected 3′ or 5′ end single‐cell data. Similar constraints also impede the investigation of spatial patterns of circRNA expression using spatial transcriptomic data. Although recent studies have enabled effective detection of circRNAs through optimized library construction protocols,^[^ ^17^ ^]^ there is an urgent need to develop efficient method for exploring circRNA using the enormous number of publicly available RNA‐seq datasets.

The integration of deep learning methods has emerged as a promising approach to address the challenges associated with modeling multicellular complexity, tissue specificity and splicing patterns.^[^ ^18^ ^]^ Notably, several splice code‐based models have successfully predicted tissue‐specific splicing regulation by estimating the probability of differential splicing between tissues.^[^ ^18c^ ^–^ ^f^ ^]^ For example, DARTS has been proven valuable for enhancing splicing analysis,^[^ ^18g^ ^]^ particularly in low‐depth sequencing data. Motivated by the success of machine learning in splicing, several studies have focused on modeling back‐splicing events using relative sequence information, such as DeepCirCode and CircDeep,^[^ ^19^ ^]^ which demonstrated the potential predictability of BSJ by distinguishing genome sequences with circRNA formation potential from others. However, these models are not applicable to predicting circRNA regulation across tissues or samples as the interaction between genomic elements and splicing regulator were not considered. Therefore, the precise prediction of circRNA regulation still remains unresolved.

Here, we developed a deep learning model, CIRI‐deep, which provides a BSJ‐read‐independent approach for predicting circRNA regulation between biological samples using total or poly(A) RNA‐seq data. The model was trained on a comprehensive dataset consisting of over 25 million high‐confidence differentially spliced or unchanged circRNA events across 397 human tissue samples. By combining both cis and trans features related to circRNA biogenesis regulation, the model achieved high accuracy when evaluated on independent datasets. Moreover, we demonstrated a significant improvement in the analysis of differentially spliced circRNAs, particularly when applied to samples with extremely low sequencing depth. To gain insights into the importance of individual features in predicting sample‐specific circRNA regulation, we employed an adapted integrated gradients method that enabled us to identify the contribution of cis and trans feature separately. When applied to single‐cell and spatial transcriptomic datasets where BSJ‐read information was unavailable, CIRI‐deep model provided valuable findings regarding the cell type and spatial heterogeneity of circRNAs that cannot be directly discerned from the original datasets alone. Overall, CIRI‐deep provides a new solution for regulated circRNA inference in low‐depth or poly(A) selected data, which largely broadens the horizon of circRNA research.

## 2 Results

### 2.1 Overview of the CIRI‐Deep Models and Data Collection

**Figure 1:**
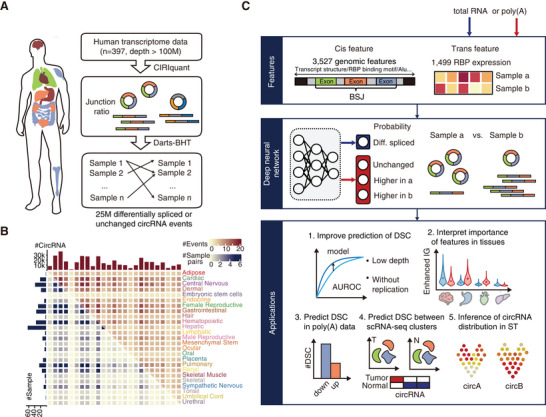
Deep learning‐based differentially spliced circRNA (DSC) events prediction model and its applications. A,B) Overview of training data for CIRI‐deep. We collected 397 human RNA‐seq data (total RNA, sequencing depth > 100 M) from circAtlas and RNA Atlas and applied CIRIquant to quantify the junction ratio of circRNAs of each sample. Each sample pair is analyzed by DARTS BHT to generate high‐confidence differentially or unchanged spliced circRNA events. Number of samples and circRNAs of each tissue, sample pairs and events between each tissue pair are shown in the heatmap. C) Schematic framework of CIRI‐deep. CIRI‐deep is trained on cis features of circRNAs and RBP expression of sample pairs (total RNA or poly(A)‐enriched RNA) through a deep neural network. Outputs of CIRI‐deep trained on total RNA RBP expression level and poly(A) selected data RBP expression level are probability of the circRNA being differentially spliced and probability of the circRNA being unchanged, of higher junction ratio in sample a, or of higher junction ratio in sample b, respectively.

Total RNA‐seq is commonly used for circRNA detection, which has an advantage over poly(A) selected RNA‐seq for comprehensive sequencing of both circRNAs and their linear counterparts. To obtain unbiased training data for our deep neural network model, we first collected total RNA‐seq data from 571 human tissue samples or cell lines from RNA Atlas and circAtlas,^[^ ^2^, ^20^ ^]^ and applied a read count threshold of 100 million (Figure S1A, Supporting Information) to filter out low‐depth datasets, resulting in the selection of 397 samples representing 25 organ systems (Table S1, Supporting Information) (**Figure** **1**A,B). We identified 75281 exonic circRNAs for training and quantified their junction ratios in all of the 397 samples using CIRIquant.^[^ ^10e^ ^]^ To generate labels for the training data, we next applied DARTS BHT to all possible pairwise comparisons of the samples,^[^ ^18g^ ^]^ which determined the significance of junction ratio difference for each circRNA event using BSJ and forward‐splicing junction (FSJ) read counts. Approximately 25 million differentially spliced (*p*(*|Δjunction ratio|* > 0.05) > 0.9) or unchanged circRNA events (*p*(|*Δjunction ratio*| > 0.05) < 0.1) were identified with high confidence (Figure 1B).

To predict the differentially spliced circRNA events between samples, we developed a deep neural network model CIRI‐deep (Figure S1B, Supporting Information), on the above data to output the probability of circRNAs being differentially spliced by incorporating both cis and trans features (Table S2, Supporting Information). Specifically, we collected 3527 relevant cis sequence features specific to each circRNA, including transcript structure, RBP binding motifs, Alu sequence, as well as other elements that have been proved to regulate canonical and back‐splicing. Additionally, we incorporated trans features by considering the expression of 1499 RBPs that are unique to each sample. These RBPs include known circRNA biogenesis‐related genes, splicing factors and RNA degradation enzymes, etc. For the poly(A) selected data, an adapted version of CIRI‐deep was trained using the corresponding RBP features paired with labels derived from the total RNA‐seq data (Figure 1C). It is important to note that the retrained model, based on poly(A) data, was adapted to output three probabilities: unchanged, a higher junction ratio in sample A or sample B. This is because poly(A) selected data usually contain much fewer BSJ reads than total RNA‐seq data, making it challenging to directly determine the higher side in a junction ratio comparison. In addition to poly(A) selected bulk RNA‐seq data, the adapted CIRI‐deep model can also be applied to infer differentially spliced circRNA (DSC) events in other data lacking BSJ‐read information, such as RNA‐seq data from 10X single cell or spatial transcriptomics (Figure 1C). The adapted CIRI‐deep model was denoted as CIRI‐deepA in the succeeding paragraphs. CIRI‐deep models take ≈60 seconds to predict ≈10 K differentially spliced circRNA events on a single CPU core (Figure S1C, Supporting Information).

### 2.2 CIRI‐Deep can Accurately Predict DSC Events between Samples

**Figure 2:**
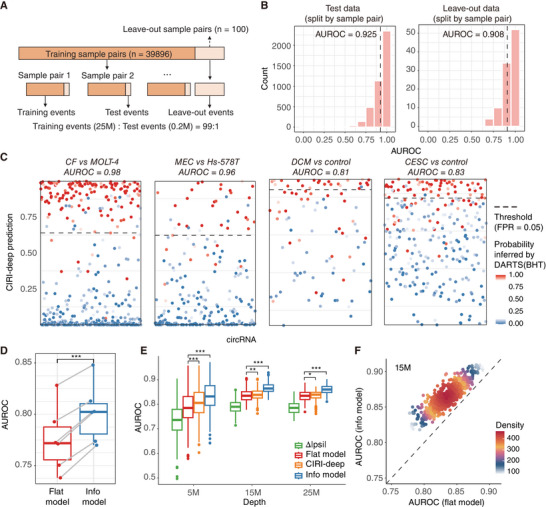
CIRI‐deep accurately predicts differentially‐spliced circRNA. A) In total, 39896 sample pairs are used for training CIRI‐deep, and 100 sample pairs are split out as leave‐out sample pairs. For each sample pair in training sample pairs, 1% of the events are split out as test events. B) Performance on test data (left) and leave‐out data (right). For test data, sample pairs with more than 10 test events are plotted. Y‐axis and X‐axis represent number of sample pairs and AUROC in each sample pair. AUROC for whole test events and leave‐out events are labeled and plotted as dash‐line. C) Generalization on leave‐out sample pair (left 2) and public data (right 2). Each dot represents differentially spliced probability predicted by CIRI‐deep for circRNA expressed in both samples. CF: conjunctival fibroblast; MEC: mammary endothelial cell; DCM: dilated cardiomyophathy; CESC: Cervical squamous cell carcinoma and endocervical adenocarcinoma. D) The performance of statistical inference combined with (Info model) or without (Flat model) CIRI‐deep in cervical cancer datasets. Ground truth are from sample pairs with replicates. *P*‐value was calculated using t‐test, ***p < 0.001. E) Performance of absolute value of *Δ|psi|*, flat model, CIRI‐deep only and info model in predicting circRNA events between sample pairs of different depth (5 M, 15 M, 25 M). P‐values was calculated using t‐test, *p < 0.05, **p < 0.01, ***p < 0.001. F) Performance of flat model and info model in 600 15 M sample pairs.

During the training process of CIRI‐deep, 99% of the labeled events were randomly extracted from each sample pair for training, while the remaining 1% were kept as test data (0.2 million events). For independent evaluation, we also kept 100 unseen sample pairs as leave‐out data (**Figure** **2**A). After eight epochs, CIRI‐deep reached an AUROC plateau of 0.925 on test data, which outperformed other machine learning methods (Figure S2A,B, Supporting Information). Meanwhile, the performance on leave‐out data also achieved an average AUROC of 0.908, with 86% of the sample pairs reaching an AUROC above 0.85 (Figure 2B). When applied to sample pairs with high variance (e.g., different tissues or different cell lines from the same tissue), CIRI‐deep showed good performance in predicting DSCs (Figure 2C, left two panels). To further test the applicability of CIRI‐deep in comparing different biological conditions within the same tissue, we applied the model to two untrained public datasets: heart samples from dilated cardiomyophathy patient (GSE162505), and cervical cancer tissue (GSE173112), along with their corresponding healthy controls (Figure 2C, right two panels). Although the training data did not contain these two samples or any other similar pathological conditions, our model generalized well to both datasets, achieving an AUROC of 0.81 and 0.83, respectively (Figure 2C; Figure S2C, Supporting Information).

One important application of our model is the inference of DSC events between sample pairs with limited coverage or lacking biological replication. To improve the identification of DSC events, the probability generated by CIRI‐deep based on RBP expression and cis sequence features was integrated into a Bayesian hypothesis statistical model as a prior probability,^[^ ^18g^ ^]^ and the posterior probability of differential splicing is computed by incorporating observed junction read counts. To evaluate the effectiveness of this approach, we used the cervical cancer tissue datasets (3 cervical cancer tissue samples vs. 2 healthy controls) for testing, with high‐confidence differentially spliced or unchanged circRNA events from the comparison with replicates as the ground truth. After incorporating CIRI‐deep (info model), the average AUROC across all six comparisons (3 × 2) increased by 3.4% compared to the inference using only BSJ and FSJ reads (flat model) in absence of biological replication (Figure 2D). In addition, we tested applicability of CIRI‐deep in detecting DSC events using low‐depth sub‐sample pairs (5, 15, and 25 million reads) randomly extracted from the cervical tissue samples. As shown in Figure 2E and Figure 2F, DSC inference combined with CIRI‐deep model outperformed the inference derived solely from junction read counts in 93.6%, 100%, and 100% of the 5, 15, and 25 million sub‐sample comparisons, with an average AUROC increase of 6.1%, 3.7%, and 3.2%, respectively. Notably, the improvement by CIRI‐deep was most prominent in the 5 million sequencing data, as the statistical test's power, without the incorporation of prior probability, was largely limited by BSJ and FSJ read counts in these low‐depth sub‐samples.

### 2.3 Model Interpretation Revealed Tissue‐Specific cis and trans Features

**Figure 3:**
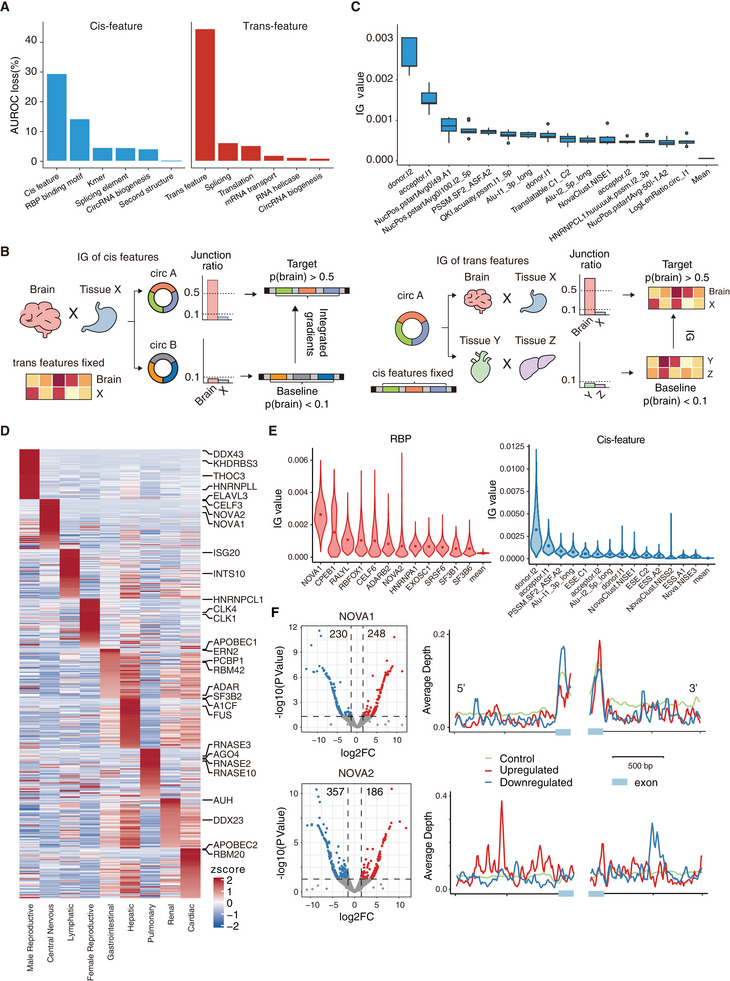
Tissue‐specific features contribute to tissue‐specific prediction. A) AUROC loss (%) of model trained by total RNA datasets with permuted cis and trans features. Splicing, translation, mRNA transport and RNA helicase related RBPs are collected from GO database. B) Workflow to identify significant tissue‐specific cis and trans features with adapted integrated gradients (AIG), taking central nervous system as example. C) IG values of top 15 cis features of common important contribution in prediction. D) IG values of top 50 RBPs (scaled by row) significant in 9 tissues. Part of tissue‐specific RBPs are labeled on right. E) Top 12 splicing‐related RBPs and cis features significant in central nervous system. Y‐axis represents IG value calculated from each group of baseline dots and target dots. F) Regulated circRNAs after *Nova1* and *Nova2* knocked out in mouse brain (left). Enrichment pattern of *Nova1* and *Nova2* surrounding up‐regulated, down‐regulated and unregulated circRNAs (right).

To interpret the contribution of features in the CIRI‐deep model, we first estimated the permutation feature importance by evaluating the AUROC loss caused by different feature groups (Table S2, Supporting Information). In the permutation test, we permutated trans features across all training samples and cis features across all circRNAs. As shown in **Figure** **3**A, the permutation of trans and cis features resulted in 44.6% and 29.5% of AUROC loss, respectively, indicating that both feature types are indispensable in the CIRI‐deep model, with sample‐specific trans features exhibiting greater importance. Interestingly, the most important feature group among the cis features was the RBP binding motif, which accounted for a 14.3% AUROC loss. In addition, by calculating the correlation between the expression level of RBPs and the junction ratio of circRNAs, we found that 11.3% (n = 170) RBPs were positively correlated (Pearson's r > 0.3, P‐value < 0.05) with more than 100 circRNAs and 34.8% RBPs (n = 521) were negatively correlated with more than 100 circRNAs, highlighting the contribution of RBP expression levels to the identification of differentially spliced circRNAs. For example, the expression of QKI is positively correlated with junction ratio of 1070 circRNAs, and these circRNAs showed significantly different levels on 115 cis features including the density of QKI binding motif (Figure S3A, Supporting Information) in flanking introns compared with other circRNAs (t‐test with Bonferroni correction, n = 6453, FDR < 0.05), indicating that cis features can contribute to DSC identification in a context‐dependent way. When considering the 44.6% AUROC loss attributed to RBP expression in the trans features, it further underscores the importance of RBPs in circRNA regulation. Known cis‐features related to circRNA biogenesis accounted for only a 5.3% AUROC loss, indicating that circRNAs are regulated through a more intricate mechanism than previously thought. Moreover, as previously shown by Xu et al.,^[^ ^21^ ^]^ splicing amount plays important role in identification of DSCs by CIRI‐deep. AUROC loss caused by disturbance of splicing amount has reached 3.95%.

To investigate the tissue‐specific regulation of circRNAs, we introduced an Adapted Integrated Gradients (AIG) approach to calculate the contribution of cis and trans features in nine organ systems (male reproductive system, central nervous system, lymphatic system, female reproductive system, cardiac system, gastrointestinal system, pulmonary system, hepatic system and renal system). As illustrated in Figure 3B, for the AIG analysis of cis features, we collected circRNAs with high junction ratio only in target tissue and circRNAs with low junction ratio in both tissues, which vary dramatically in prediction results. To identify cis features contributing to the differential splicing, the integrated gradient value (IG value) for each cis feature was calculated between two groups of circRNAs as metrics. As we calculated IG value between same sample comparisons, trans features keep constant and have no effect on evaluation of cis features. For the analysis of trans features, the IG value was calculated similarly (Figure 3B, Methods).

We next applied AIG to each of the nine organ systems, and observed substantial variation in the numbers of specific features identified across these tissues (Figure S3B,C, Supporting Information). Notably, the male reproductive system and central nervous system exhibited the highest numbers of enriched specific features, with ≈200–300 specific cis and trans features each. In contrast, only 31 and 23 cis and trans features were identified as specific for pulmonary system. Despite variations in cis feature specificity, the top 15 cis features were consistently found to contribute significantly to the prediction of various tissues (Figure 3C). Among these features, donor and acceptor strength of back‐splicing sites were most important, surpassing the importance of the donor and acceptor of adjacent exons. Additionally, the presence of Alu sequences surrounding back‐splicing sites was also highly related to circRNA junction ratio, which is consistent with previous reports (Figure 3C).^[^ ^4^, ^6^ ^]^ Compared with cis features, trans features showed higher specificity (Figure 3D; Figure S3D,E, Supporting Information). For example, *KHDRBS3*, a testis‐specific feature that significantly contributes to DSC prediction, is a splicing factor with extremely high abundance in testis and was found to increase circRNA stability.^[^ ^22^ ^]^ *FUS*, a known circRNA regulator, exhibited relatively low abundance in the liver, making it a liver‐specific feature with a negative contribution.^[^ ^23^ ^]^ RNA editing enzymes *APOBEC1* and *APOBEC2* were among the top significant features in the gastrointestinal and cardiac system, suggesting their potential influence on circRNA biogenesis similar to *ADAR*. These trans features with high IG value variance also tended to have tissue‐specific expression (Figure S3F, Supporting Information). Since splicing amount strongly influenced the identification of differentially spliced circRNAs, we further investigated the relationship between splicing amount and tissue‐specific circRNAs. It turns out that splicing amount has a weaker correlation with the junction ratio of tissue‐specific circRNAs in eight out of the nine organ systems (Figure S3G, Supporting Information).

Recent studies have shown that circRNAs are widely distributed in neuronal tissues, which encouraged us to investigate brain‐specific cis and trans features for biological insights. As shown in Figure 3E, we observed that the most critical splicing‐related features in the brain included *NOVA1* and *NOVA2*, two brain‐specific splicing factors. Meanwhile, *NOVA*‐related RBP binding motifs were found to be important cis features specific to the brain (Figure S3H–J, Supporting Information). To further investigate the impact of *NOVA1* and *NOVA2* on circRNA expression, we analyzed a publicly available transcriptomic dataset (GSE69711), where *Nova1* and *Nova2* were knocked out in the mouse brain. Our findings revealed alterations in the expression levels of numerous circRNAs compared to control samples, indicating that both *Nova1* and *Nova2* exert binary effects on circRNA expression. Next, we compared the average coverage of binding sites around upregulated and downregulated circRNAs using HITS‐CLIP data. The enrichment patterns of upregulated and downregulated circRNAs seemed to be asymmetrical. Specifically, *Nova1* binding regions were predominantly located in the downstream exon of upregulated circRNAs and the upstream exon of downregulated circRNAs; *Nova2* binding regions tended to occur in the downstream intron of downregulated circRNAs and the upstream intron of upregulated circRNAs (Figure 3F). Previous studies have shown that splicing outcomes heavily rely on RBPs and their binding positions.^[^ ^24^ ^]^ The differential binding regions of *Nova1* and *Nova2* suggest that they may exhibit distinct regulatory and positional effects on circRNA biogenesis.

### 2.4 CIRI‐DeepA is able to Predict DSC Events from Bulk and Single Cell Poly(A) selected RNA‐seq Data

**Figure 4:**
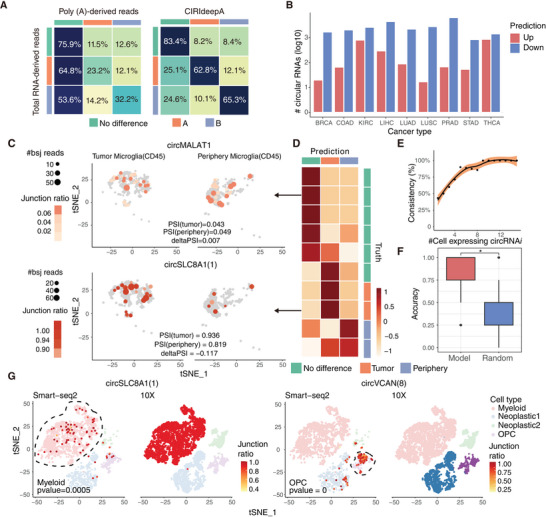
Prediction of differentially spliced circRNA between scRNA‐seq clusters. A) The heatmap is a multiclass confusion matrix of 3 categories: no difference, higher junction ratio in sample A and higher junction ratio in sample B. Each row represents circRNAs identified as no difference, higher in A or higher in B with gold standard total RNA‐seq datasets. Each column represents the percentage of circRNAs classified into three categories in each row with poly(A)‐derived reads or CIRI‐deepA, respectively. B) Number of circRNAs predicted to be up‐ or down‐regulated in tumor samples compared with pairwise control from TCGA datasets. CircRNAs are identified as up‐ or down‐regulated if their junction ratios are predicted to be higher in tumor or pairwise control in more than 35% sample pairs. C) Two high‐confidence differentially spliced events between glioma microglia and periphery microglia in Smart‐seq2 datasets (GSE84465). Cells with the circRNA detected are labeled, with size and color indicating number of back‐splicing reads and junction ratio of the circRNA in the cell. D) Prediction probability of 10 high‐confidence differentially spliced events between a certain cell‐type clusters in tumor and periphery tissue (*p* > 0.9 or *p* < 0.1, number of cells in tumor or periphery tissue > 10). E) Consistency (%) between statistical inference (DARTS BHT) from Smart‐seq2 data and model prediction from 10X scRNA‐seq data for circRNA events detected in different number of cells. F) Boxplot of prediction accuracy of 8 high‐confidence marker circRNAs. *P*‐value was calculated using t‐test, n = 8, *p < 0.05. G) Prediction of marker circRNA in 10X scRNA‐seq datasets. Smart‐seq2 glioma (GSE84465) and 10X glioma (GSE131928) datasets are merged and 4 common clusters (Myeloid, Neoplastic 1, Neoplastic 2 and OPC) are included. Marker circRNAs are tested through Wilcoxon rank test (*p* < 0.05, number of circRNA expressing the circRNA > 5) in Smart‐seq2 datasets, and clusters with higher marker circRNA junction ratio are highlighted by dashed line. Prediction of marker circRNAs is made to compare junction ratios between cells in each cluster and the rest cells in 10X datasets. Clusters predicted with higher junction ratio are highlighted.

An important application of CIRI‐deep is the prediction of DSC from poly(A) selected RNA‐seq data, which primarily captures mRNA but lacks circRNA reads (Figure 1C). We developed an adapted version of CIRI‐deep, called CIRI‐deepA, which was trained on RBP expression from the poly(A) data in RNA Atlas using a three‐class classification scheme (see Methods). To assess the performance of CIRI‐deepA, we randomly selected 15 unseen samples as leave‐out data and identified 62127 DSC events in the corresponding total RNA‐seq data for testing. As expected, the detection of BSJ reads in poly(A) data revealed only a small subset of circRNA events (4.8%) compared to those in the corresponding total RNA‐seq data (Figure S4A–C, Supporting Information), and only 28.3% of the DSC events from the subset could be identified by traditional statistical tests. In contrast, by employing CIRI‐deepA, we could analyze the full set of 62127 circRNA events and accurately predict the majority of DSC events, achieving an average AUROC of 0.868 (**Figure** **4**A; Figure S4D, Supporting Information). The high sensitivity of CIRI‐deepA enables the analysis of DSC events from large cohorts of poly(A) data. For example, we applied CIRI‐deepA to tumor and normal control samples from the TCGA dataset, and detected 899–6498 regulated circRNAs across nine cancer types. As shown in Figure 4B, a higher number of down‐regulated circRNAs were predicted compared to up‐regulated circRNAs in all cancer types, consistent with previous reports on cancer profiling.^[^ ^25^ ^]^

At single‐cell resolution, circRNAs can be sporadically detected by full‐length scRNA‐seq methods such as Smart‐seq2.^[^ ^16b^ ^]^ To evaluate the performance of our model in predicting DSC events at the single‐cell level, we downloaded Smart‐seq2 scRNA‐seq data (GSE84465) from glioma and peripheral tissues, and profiled circRNA expression in different cell clusters using CIRIquant. The ten most abundant circRNAs differentially spliced or unchanged between clusters in tumor and healthy tissues (Figure 4C,D; Figure S5, Supporting Information) were identified using a previously described statistical model,^[^ ^18g^ ^]^ which served as validation for CIRI‐deepA predictions. As shown in Figure 4D, CIRI‐deepA accurately predicted nine of the ten events, including two events higher in glioma, two events higher in peripheral tissue, and five unchanged events, demonstrating the high accuracy of CIRI‐deepA predictions. It should be noted that circRNAs were usually detected by CIRIquant in only a small fraction of cells with limited BSJ read counts, and such a sparsity and low coverage can hinder the downstream identification of DSC events by the statistical test. As expected, we observed that the consistency for 67 DSC events between CIRI‐deepA and the statistical test declined from nearly 100% to 40% (Figure 4E), suggesting that CIRI‐deepA should have broader applicability due to its ability to predict DSC events independent of BSJ reads.

Droplet‐based single‐cell platforms such as 10x Genomics have been widely used for gene‐level quantification, which only sequence the 3′ or 5′ end of RNA transcripts and therefore cannot be directly used for circRNA detection. To assess the feasibility of using CIRI‐deepA with droplet‐based scRNA‐seq data, we applied it to a 10X glioma dataset (GSE131928) to infer cluster‐specific circRNAs defined in the aforementioned Smart‐seq2 dataset. As shown in Figure 4F, the accuracy of CIRI‐deepA in predicting differential splicing for eight circRNA markers was significantly higher than that of a null model. For example, our model successfully predicted circSLC8A1(1) as a myeloid‐specific circRNA with minimal expression in the OPC, Neoplastic1, and Neoplastic 2 clusters, consistent with the findings based on Smart‐seq2 data (Figure 4G). Another circRNA marker, circVCAN(8), was detected in 105 (27.0%) cells of the OPC cluster but not in any myeloid cells. While CIRI‐deepA successfully predicted the DSC event between the OPC and myeloid clusters, it also successfully predicted circVCAN(8) to have a higher junction ratio in Neoplastic 1 cluster, which showed sporadic detection in Smart‐seq2 data compared to the other two clusters.

### 2.5 Application in Spatial Transcriptomics

**Figure 5:**
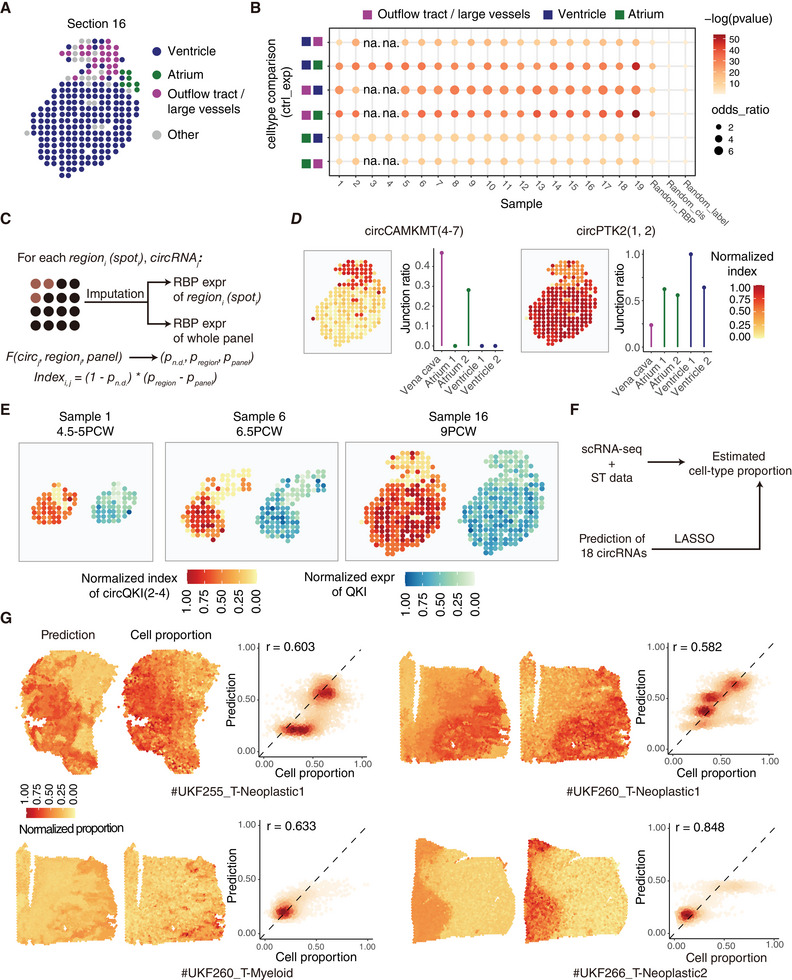
Application in spatial transcriptomics. A) The fetal heart ST panel is split into 4 anatomical regions, taking tissue section 16 as example (EGAS00001003996). B) CIRI‐deepA predicted region specific circRNAs between different regions are enriched in bulk data (RNA Atlas) derived result (fisher exact test). Dot size and color indicate ratio and *‐log(pvalue)*. Random_label: randomly chosen circRNAs (same size of region specific circRNAs in bulk data). Random_RBP: prediction using randomly permutated RBP expression value as input. Random_cis: prediction using randomly permutated cis features as input. C) Workflow for calculating circRNA index for each region or spot. F denotes the CIRI‐deepA model. D) CircRNA relative region index plot of section 16 (left) validated by junction ratio in corresponding bulk data (right). Vena cava, atrium 1, atrium2, ventricle1 and ventricle 2 are corresponded to outflow tract/large vessels, atrium and ventricle regions, respectively. E) Normalized circRNA index of circQKI(2‐4) and normalized expression of QKI in section 1, 6 and 16. F) Workflow for fitting the cell‐type proportion with LASSO using prediction probability of 18 circRNA as input (probabilities of higher in sample A and higher in sample B). The 18 circRNAs are manually selected for low correlation of prediction probability. G) Predicted cell‐type abundance and deconvolution (CARD)‐derived cell‐type abundance in 4 samples. Pearson correlation coefficient is computed between output of Lthe LASSO model and deconvolution result.

Spatially resolved transcriptomics (ST) offers valuable insights into cellular organization and interaction; however, the detection of circRNAs based on BSJ reads from spatial transcriptomic data remains a significant challenge. As a BSJ‐read‐independent prediction model for DSC events, CIRI‐deepA presents a promising opportunity for circRNA analysis in ST data. To evaluate the capability of CIRI‐deepA in predicting region‐specific circRNAs, we applied it to three sub‐regions (i.e., ventricle, atrium and outflow tract/large vessels) of a developing human heart dataset (EGAS00001003996) based on imputed RBP expression (**Figure** **5**A). CIRI‐deepA successfully discriminated 1088–2618 sub‐region specific circRNAs in comparisons among the sub‐regions, out of 23459 circRNAs identified in heart tissue (data from RNA Atlas). Using the sub‐region‐specific circRNAs derived from similar histological regions (i.e., vena cava, ventricle and atrium) of RNA Atlas as a reference (Figure S6, Supporting Information), the DSC events identified by CIRI‐deepA showed a strong enrichment for region‐specific circRNAs, with *p*‐values ranging from 3.5 × 10^−54^ to 3.7 × 10^−6^ (Fisher's exact test) and odds ratios ranging from 2.07 to 6.87. In contrast, randomly selected circRNAs or events predicted from randomly permuted RBP expression and cis features showed weak or no enrichment (Figure 5B). When using DSC events (|*delta junction ratio*| > 0.05) from RNA Atlas between sub‐regions as a reference, CIRI‐deepA predictions showed a significantly higher accuracy compared with randomly selected circRNAs in all six pairwise comparisons (Figure S7, Supporting Information). For example, the top 100 accuracy of predicted DSC events ranged from 0.72 to 0.87, but only 0.17 to 0.35 for randomly selected circRNAs. We also observed that CIRI‐deepA achieved higher accuracies for the top‐5, top‐10 and top‐20 predictions of DSC events between the outflow tract and ventricle/atrium region compared to those between the ventricle and atrium, potentially due to the high similarity of cell types between the atrium and ventricle.

In addition to predicting pairwise DSC events between regions, it would be valuable to generate a relative junction ratio map for each circRNA, highlighting regions or spots with higher ratios. To achieve this, we devised a circRNA index that indicates the relative junction ratio in each region or spot. This index was derived, from three probabilities of differential splicing output by CIRI‐deepA, which takes into account imputed RBP expressions in both the region and the pseudo‐bulk mixture of the entire ST sample (Figure 5C) (see Methods). The circRNA index profile demonstrated a high consistency among similar regions (Figure S8A–C, Supporting Information). For example, the Pearson's correlation coefficient between trabecular ventricular myocardium and compact ventricular myocardium was calculated to be 0.991. In contrast, the correlation coefficients between ventricle/atrium and outflow tract were 0.871 and 0.840, respectively, indicating greater variability in circRNA expression across these regions.

To assess the reliability of relative junction ratio map based on the circRNA index, we investigated 50 circRNAs with the highest deviation across different regions. Notably, we observed a significant rank correlation between the indices of these circRNAs in three sub‐regions (ventricle, atrium and outflow tract) and the corresponding bulk tissues (Figure S9A, Supporting Information, *p‐value* = 0.0001). In a more specific case, circCAMKMT(4‐7) showed the highest index at both region and spot resolution in the outflow tract region, and it also showed the highest junction ratio in bulk vena cava tissue. In contrast, circPTK2(1, 2) junction ratios were elevated in the ventricle region instead of the outflow track region (Figure 5D; Figure S8D, Supporting Information), consistent with the observations in bulk tissues. Previous studies have demonstrated that both circQKI(2‐4) and mRNA *QKI* play important roles in human myogenesis.^[^ ^3b^ ^]^ In our circRNA index plot, we found that circQKI(2‐4) consistently showed relatively high junction ratios in the ventricle and atrium regions compared to the outflow tract region throughout the heart development stages (Figure S9C, Supporting Information). This aligns with the junction ratio profile in bulk tissue samples (Figure 5E; Figure S9B, Supporting Information). Similarly, the host gene *QKI* showed the highest expression in the ventricle and atrium regions (Figure 5E), suggesting that circQKI(2‐4) and *QKI* may both contribute to myocardial development in the human heart.

To assess the capability of our model in deconvolving cell type in spatial transcriptomic data, we applied CIRI‐deepA to a glioma ST dataset comprising 28 samples that were sequenced using 10X Visium, and then outputted predicted probabilities of circRNAs differentially spliced across spots. We selected 18 circRNAs that were identified with high cell type specificity (Figure S10, Supporting Information) but low correlation of prediction probabilities with each other, and then used their probabilities to train three independent LASSO models capable of predicting the proportion of the three most abundant cell types (Neoplastic1, neoplastic2, myeloid) in each spot (Figure 5F). As shown in Figure 5G, the cell type proportions predicted by these LASSO models were highly consistent with those deconvoluted by CARD.^[^ ^26^ ^]^ Notably, the proportion of the predominant neoplastic1 cell type in UKF255 and UKF260 exhibited Pearson correlation coefficients of 0.603 and 0.582, respectively, between CARD and our LASSO model. The LASSO models also effectively captured the distribution of the less abundant cell types, neoplastic 2 and myeloid, showing correlations of 0.633 and 0.848, respectively, with CARD deconvolution (Figure 5G). In summary, these results demonstrated the promising ability of CIRI‐deepA to identify region‐specific circRNAs.

## 3 Discussion

In this study, we developed a deep learning model, CIRI‐deep, to predict differentially spliced circRNA events between biological samples. CIRI‐deep was extensively trained on a comprehensive dataset consisting of 397 human tissue samples from RNAAtlas and circAtlas.^[^ ^2^, ^20^ ^]^ It exhibited superior performance on both test and independent datasets, surpassing traditional methods rely on back‐splicing junction reads, particularly on datasets with limited circRNA identification, such as low‐depth or poly(A) selected RNA‐seq data. CIRI‐deep model also offered new insights into circRNA regulatory mechanisms. By incorporating adapted integrated gradients, we were able to quantify the importance of tissue‐specific cis and trans features. Furthermore, we evaluated the applicability of our model in inferring cluster‐ and region‐specific circRNAs in single‐cell and spatial transcriptomic data. These findings underscore the promising potential of employing deep learning models for circRNA analysis, opening avenues for further exploration in this field.

CIRI‐deep and CIRI‐deepA were introduced to achieve different goals. Until recently, identification and quantification of circRNAs were generally based on the detection of BSJ reads, which requires deep sequencing transcriptomic data.^[^ ^11b^ ^]^ This is due to the relatively low expression levels of circRNAs and the inherent difficulty in detecting BSJ reads compared to mRNA reads distributed across the entire transcript. To overcome the limitations imposed by sequencing depth, we developed CIRI‐deep to infer differentially spliced circRNAs and provide informative predictions independent of back‐splicing junction reads. Combined with the Bayesian Hypothesis Testing (BHT) statistical model,^[^ ^18g^ ^]^ CIRI‐deep significantly improves the analysis of differentially spliced circRNAs in samples with low sequencing depth. On the other hand, the widely used poly(A) selection step in cDNA library construction is believed to preferentially remove RNA transcripts lacking polyadenylated tails. Our evaluation corroborated the unsuitability of poly(A) selected RNA‐seq data for direct identification of differentially spliced circRNAs due to both low sensitivity of BSJ read detection and the bias toward unchanged events (Figure 4a; Figure S4, Supporting Information). To this end, CIRI‐deepA provided a promising solution for comprehensive identification of differentially spliced circRNAs, which also enabled circRNA analysis with prevalent large project employing poly(A) selected RNA‐seq, such as GTEx and TCGA.^[^ ^27^ ^]^

Although recent studies have used full‐length scRNA‐seq to explore single‐cell level circRNA expression,^[^ ^16b^ ^]^ most single cell and spatial transcriptomic approaches that are based on 3′ or 5′ end sequencing cannot be used for further circRNA analysis. Utilizing the deep learning algorithm, CIRI‐deepA for the first time provides an efficient method for profiling circRNA splicing output with poly(A) selected datasets at the single cell or spatial level. Through our evaluation, CIRI‐deepA exhibited its efficiency in inferring differentially spliced circRNAs between single‐cell clusters, which significantly enhances our ability to analyze regulated circRNA events occurring during critical processes such as tumorigenesis or embryonic development (Figure 4). Meanwhile, we showcased that CIRI‐deepA predictions can be used to reconstruct spatially resolved maps of circRNA junction ratios, which to our knowledge cannot be computed directly from currently available spatial transcriptomic data (Figure 5). Remarkably, the probabilities predicted by CIRI‐deepA for circRNA events with high cell type specificity align well with the task of predicting cell type proportions for each spatial spot. While traditional spatial transcriptomic analysis relies on thousands of marker genes to infer cell type proportions, our study demonstrates that accurate cell type proportions can be inferred using only a limited number of representative circRNAs. This suggests the potential application of cell type‐specific circRNAs in single‐cell transcriptomic analysis (Figure 5).

Given that circRNAs are expressed with high specificity,^[^ ^2^, ^20^ ^]^ it is reasonable to assume that the regulation of circRNA biogenesis, which remains incompletely understood, varies extensively across different tissues.^[^ ^28^ ^]^ Exploring the features on which the model relies to accurately predict tissue‐specific circRNAs can offer new insights into the regulation of circRNAs.^[^ ^29^ ^]^ In a previous study, Jha et al.^[^ ^30^ ^]^ systematically evaluated and demonstrated the applicability of enhanced integrated gradient (EIG) in identifying significant features that contribute to the prediction of tissue‐specific splicing regulation in the splicing code model. Given that our model incorporates both trans and cis features, we adapted EIG to assess the contribution of these features independently in each trial and avoid potential interference between them. This approach enabled us to characterize significant cis and trans features that contribute to the prediction of tissue‐specific regulation of circRNA biogenesis. Notably, many of the identified significant features have previously been demonstrated to play important roles in circRNA regulation.^[^ ^4^, ^6^, ^22^, ^23^ ^]^ Meanwhile, the interpretation of CIRI‐deep model suggests that the preference for RBP usage in circRNA biogenesis vary widely across tissues, which partially explained the underlying mechanism of tissue‐specific circRNA expression profile. Several tissue‐specific RBPs, such as *NOVA1*, *NOVA2* and *APOBEC1*, emerged as potential circRNA regulators, which may guide experimental exploration on circRNA biogenesis.

Although CIRI‐deep and CIRI‐deepA can be applied to predict differentially spliced circRNAs in low‐depth RNA‐seq data, the performance decreases when the sequencing depth is too low to accurately quantify RBP expression levels. As shown in Figure 2E, the AUROC of CIRI‐deep to identify differentially spliced circRNA is lower for samples with a depth of 5 million than for samples with a depth of 15 and 25 million. For samples with extremely low sequencing depth, such as spatial transcriptomic data, we recommend performing gene expression level imputation before prediction. Meanwhile, since the training of CIRI‐deep models relies on cis‐features from the reference genome, the prediction of cancer‐specific circRNAs expressed due to genomic mutation or RBP dysregulation may be beyond the scope of our tools.

## 4 Conclusion

In summary, the introduction of CIRI‐deep represents a promising solution for inferring differentially spliced circRNAs in various types of datasets. The model effectively addresses the challenges posed by low sequencing depth or poly(A) selected data, as well as single‐cell and spatial transcriptomic data, significantly broadening the horizons of circRNA research. We believe that the versatility and robust performance make it valuable tool for researchers exploring the regulatory mechanisms and functional implications of circRNAs across different experimental settings.

## 5 Experimental Section

### CIRI‐Deep and CIRI‐deepA Model for Predicting Differentially Spliced circRNAs

The CIRI‐deep and CIRI‐deepA models were designed for predicting the differentially spliced circRNAs between biological samples using total RNA‐seq data and poly(A) selected data, respectively. Both models were trained using cis sequence features and sample‐specific trans features as described below. CIRI‐deep outputs the probability of a given circRNA being differentially spliced between two biological samples, and CIRI‐deepA outputs three probabilities that sum to one indicating: unchanged, a higher junction ratio in sample A or sample B.

The overall architecture of CIRI‐deep and CIRI‐deepA were shown in Figure 1 and Figure S1 (Supporting Information). The basic unit of CIRI‐deep and CIRI‐deepA is a dense block, which consists of four layers including a fully connected layer, a batch normalization layer, rectified linear unit (ReLU) and a dropout layer. CIRI‐deep and CIRI‐deepA have four dense blocks, with input size of 1200, 500, 300, and 200, respectively. To address the potential overfitting problem and to improve the generalization ability of the models, we added dropout layers that randomly drop connections at rates of 0.5, 0.3, 0.2, and 0.1,^[^ ^31^ ^]^ respectively. Batch normalization layers were added to accelerate the training process.^[^ ^32^ ^]^ The activation functions used in the output layers of CIRI‐deep and CIRI‐deepA were sigmoid and softmax, respectively.

### Training Data Generation

We downloaded human total RNA‐seq datasets (fastq files) from RNAAtlas and circAtlas (Table S1, Supporting Information) for training.^[^ ^2^, ^20^ ^]^ Because most circRNAs are expressed at relatively low abundance compared to their linear counterparts, 397 deep sequenced samples with more than 100 million reads were kept for accurate identification and quantification of circRNAs. Trimmomatic v0.39 were used to remove adapters and low‐quality bases with the parameters “ILLUMINACLIP:TruSeq3‐PE‐2.fa:2:30:10:2:true MINLEN:36”.^[^ ^33^ ^]^ The trimmed reads were then aligned to the human reference genome hg19 using bwa v0.7.17.^[^ ^34^ ^]^ We applied CIRIquant to identify and quantify circRNAs with GENCODE v38lift37 as annotation file.^[^ ^10e^ ^]^ CIRIquant generates a pseudo reference based on circRNAs identified by CIRI2, and realigns sequencing reads to it to output BSJ read count, FSJ read count, junction ratio, circRNA type (intronic, exonic or intergenic) and host gene for expressed circRNAs in each sample. All exonic circRNAs were used for training.

To generate training labels, we applied DARTS BHT(flat) to each pairwise sample comparison.^[^ ^18g^ ^]^ DARTS BHT is a Bayesian statistical framework designed for determining the statistical significance of differential or unchanged splicing events at a given ΔPSI threshold between biological samples. DARTS BHT can use an uninformative prior (flat model) or incorporate an empirical prior (info model) to determine significance from RNA‐seq data Here, we use predictions derived from the deep learning model as empirical prior to augment inference. Specifically, events with posterior probability *p*(|Δ*junction* *raio*| > 0.05) > 0.9 are labeled as positive and events with posterior probability *p*(|Δ*junction* *raio*| > 0.05) < 0.1 were labeled as negative. To ensure sufficient statistical power, we kept events with BSJ and FSJ read count greater than two and sum of BSJ and FSJ read count greater than 20 in both samples, resulting in 25039091 high‐confidence differentially spliced or unchanged events with a ratio of 1:3 for training. CIRI‐deepA was designed to predict the differential splicing pattern of circRNA using RBP expression derived from poly(A) selected data as input. Significant events of differentially spliced and unchanged circRNA identified from paired total RNA‐seq data from RNAAtlas are labeled as positive and negative events for training. Positive events were further labeled as “higher in A” (circRNA had higher junction ratio in sample A) or “higher in B” (circRNA has higher junction ratio in sample B).

### Training and Testing of CIRI‐Deep and CIRI‐deepA

For each sample comparison, we randomly selected 1% of the events to test and decide the early stop point, and the remaining events were used for training. To keep the test data balanced, the number of negative and positive events is equal. For independent evaluation, we also randomly selected 100 sample comparisons as leave‐out datasets.

During training, the training data was split into mini batches with size of 512. To avoid the training bias, each batch is composed of half positive events and half negative events. Binary cross entropy was used as the loss function and minimized by Adam optimizer.^[^ ^35^ ^]^ Model performance was evaluated on test data after each epoch of training using AUROC. The model was trained for eight epochs on NVDIA TITAN X GPU.

For CIRI‐deepA, 15 samples were randomly selected as leave‐out datasets for more rigorous evaluation. Categorical cross entropy was used as the loss function. The model was trained for seven epochs.

### Cis and Trans Feature Extraction

There are a total of 6525 features in CIRI‐deep, of which 3527 are cis features and 2998 are trans features. These features covered the entire collection of splicing related sequence features and RNA binding proteins previously used by Zhang et al. to predict exon skipping patterns.^[^ ^18g^ ^]^ In addition to those features, we added four classes of features related to circRNA generation. Cis features were extracted from different regions surrounding back‐splicing junctions. A1 and A2 denotes upstream and downstream BSJ exon. If circRNA is composed of a single exon, A1 and A2 refer to the same exon. C1 and C2 denote upstream and downstream exon. I1 and I2 denote upstream and downstream flanking intron.

The full feature set was listed in Table S2 (Supporting Information), which consists of 12 categories:

1. Transcript structure: Length of flanking intron, length of circexons, distance between junction sites and their ratio, and whether the circRNA will induce a frameshift.
2. Translatability: Whether transcript can be translated without stop codons (all three reading frames are tested).
3. Splicing strength: Splicing strength at junctions between C1‐A1 and A2‐C2.
4. Conservation score: Average conservation score of the first and last 100 nucleotides of I1 and I2.
5. Average and maximum second structure score at junction sites.
6. Average nucleosome occupancy score of the first and last 100 nucleotides of I1 and I2, as well as that of the first and last 50 nucleotides of A1 and A2.
7. Exon splicing enhancer and silencer, intron splicing enhancer and silencer.
8. Kmers in the first and last 150 bp of I1 and I2.
9. RBP binding motifs in I1, I2, A1, A2, C1 and C2.
10. Reverse complementary match between I1‐I2, I1‐I1 and I2‐I2.
11. Repetitive elements in I1 and I2.
12. Splicing amount.

As previously reported, repetitive elements and RBP binding motifs in flanking introns regulate circRNA generation. The collection of splicing related features only covered intron region of 150 bp outside the back‐splicing junction sites. Considering the potential long‐range regulatory effect of flanking introns, features about RNA binding motifs, repetitive elements and reverse complementary match (RCM) of the entire flanking introns were added. In previous studies, Xu and Zhang found that splicing amount was related to circRNA expression abundance.^[^ ^21^ ^]^ Features of splicing amount, defined as the total amount of back‐splicing and canonical splicing of a gene, were also added. New features were calculated as follows:

1. Intron repetitive elements: We extracted Alu/L1/L2 repetitive element sites from USCS Genome Browser Repeat Masker track. The feature is defined as number of Alu/L1/L2 repetitive elements in flanking introns.
2. Intron RBP motif: We included motif counts of 111 RBPs and PSSM scores of 14 RBP in flanking introns, and also scores normalized by intron length. PSSM matrix was downloaded from RBPmap.^[^ ^36^ ^]^
3. Reverse complementary matches: Reverse complementary matches (RCM) occurring in flanking regions facilitate circRNA generation, but may hinder circRNA generation when occurring within introns. RCM pairs between intronic sequence and reverse complementary sequence were identified using BLAST as previously described by Cortez‐Lopez et al.^[^ ^37^ ^]^ with word size 11. Counts of RCM pairs between flanking introns and within introns, and counts weighted by intron length were added to the input features.
4. Splicing amount: For each circRNA and sample, the reads spanning annotated junction in the parental gene were extracted from alignment file (SAM file). Counts per million (CPM) values of junction reads in both samples were extracted as input feature. Splicing amount was not used for CIRI‐deepA training.

Other features were calculated as described in study of Zhang et al.^[^ ^18g^ ^]^ Trans features included expression value of 1499 RNA binding proteins for both samples. After trimming, the reads were aligned to the human reference genome hg19 using Hisat2 v2.0.5.^[^ ^38^ ^]^ Gene expression levels were quantified as TPM using StringTie v1.3.5.^[^ ^39^ ^]^ All the features were normalized by the maximum absolute value across the whole training datasets.

### Adapted Integrated Gradient for Identifying Tissue‐Specific cis and trans Features

The method of Enhanced integrated gradient to quantify contribution of tissue‐specific cis and trans feature to differential back‐splicing events independently was adapted.^[^ ^30^ ^]^ Generally, contribution of each feature to target‐tissue‐specific circRNAs was determined with respect to baseline events *x’*∈*χ*. Two groups of events as target events and baseline events was selected: events with circRNA specifically generated in target tissue and events with circRNA rarely generated in tissue pairs including target tissue. Between target events and baseline events, the integral over the gradients along a linear path *γ* was calculated for each feature, which was denoted as IG value. The linear path *γ* between baseline events *x’* and target events *x* is determined as *γ(α)* = *x’* + *α*(*x – x’*), *α*∈[0, 1]. In the calculation, three baseline events that were closest to the median of baseline class (Euclidean distance) to represent the entire baseline class was selected. The IG value of the *j*th feature was calculated as follows:

(1) $$\begin{array}{ccc} IG\;value_{j}\left(x\right) & = & \int_{\alpha =0}^{1}\frac{\partial F\left(\gamma \left(\alpha \right)\right)}{\partial \gamma_{j}\left(\alpha \right)}\frac{\partial \gamma_{j}\left(\alpha \right)}{\partial \alpha }d\alpha \\ & = & \left(x_{j}-x_{j}^{\prime }\right)\times \int_{\alpha =0}^{1}\frac{\partial F\left(x^{\prime }+\alpha \times \left(x-x^{\prime }\right)\right)}{\partial x_{j}}d\alpha \end{array}$$

where *F* is the deep learning model.

AIG was applied in nine organ systems including male reproductive, central nervous, lymphatic, female reproductive, cardiac, gastrointestinal, pulmonary, hepatic and renal system. Each organ system included multiple tissue samples from RNAAtlas. The IG value of cis and trans features was evaluated for CIRI‐deepA model as follows:

1. Cis feature: For each organ system, we extracted all sample comparisons between this organ system and other organ systems. In each sample comparison, differentially spliced circRNA events with junction ratio higher than 0.5 in sample from target organ and lower than 0.1 in samples from other organs were selected as target events, while unchanged circRNA events with junction ratio lower than 0.1 in both samples were selected as baseline events. For each sample comparison, IG values were calculated for all cis features. IG values of trans features were all 0 as sample comparison was fixed in each trial. The mean absolute IG value of each cis feature across all sample comparisons was calculated as the final value for the organ system.
2. Trans feature: For each organ system, we first selected circRNAs with high junction ratio greater than 0.5 in all samples of this system. For each selected circRNA, target events were constructed by searching for sample comparisons between this organ system and other organ systems, with this circRNA being differentially spliced; baseline events were constructed by searching for sample comparisons consisting of two samples from other organ systems, with this circRNA being unchanged and have a low junction ratio < 0.1. For each circRNA, IG values were calculated for all trans features. IG values of cis features were all 0 as circRNA was fixed in each trial. The mean absolute IG value of each trans feature across all selected circRNAs was calculated as the final value for the organ system. All the computed IG values were given in Table S3 (Supporting Information).

### Evaluation of Generalization on Public Data

The generalization ability of CIRI‐deep was evaluated on five public datasets (GSE161960, GSE162505, GSE172315, GSE173112, GSE179321).^[^ ^40^ ^]^ Detailed information of these datasets is provided in Table S1 (Supporting Information). Identification and quantification of circRNAs in these datasets were carried out as mentioned above (Training data generation). Sample comparison was performed between the corresponding experimental and control sample group set in each study. Differentially spliced or unchanged events were labeled at the same threshold as for training data. All evaluations of generalization were restricted to the circRNA events constructed during training. Each input trans feature was normalized by the maximum gene expression value in the corresponding training datasets, and the normalized value exceeding 1 was reset to 1. AUROC was used for evaluation.

### Inference of DSCs in Sample Pairs Lacking Replication or with Limited Coverage

To more accurately infer differentially spliced circRNA in sample pairs lacking replication or with limited coverage, the prediction of CIRI‐deep was incorporated into the DARTS BHT framework. A dataset of cervical cancer (GSE173112) for evaluation was used.^[^ ^40d^ ^]^ The dataset consists of cervical cancer tissues and normal cervical tissues, and each group has three biological replicates collected from three cervical cancer patients and three normal patients, respectively. One normal cervical tissue sample (GSM5260031) was filtered out for the abnormal detection rate of circRNA. Then, differentially spliced and unchanged circRNAs between these two groups with replication were identified by CIRI3 (https://github.com/gyjames/CIRI3) with rMATS statistical model as ground truth, at a threshold of *p* < 0.01, *Δjunction ratio* > 0.05 and *p* > 0.99, *Δjunction ratio* > 0.05, respectively.^[^ ^41^ ^]^

CIRI‐deep was applied to the six sample comparisons (3×2) and inferred differentially spliced and unchanged circRNAs using flat and info models as described above. The significance of differential performance between flat model and info model was tested by one‐sided t‐test.

To evaluate the performance of CIRI‐deep on low depth samples, we randomly extracted 5, 15, and 25 million reads pairs from each sample with 10 replicates in the cervical datasets, and performed 600 sample comparisons between cancer and normal groups for each depth. The performance of inference using |*Δjunction ratio*| of events, probability derived from flat model, prediction value of CIRI‐deep and probability derived from info model were then evaluated.

### Permutation Test for Feature Group Contributions

To evaluate the contribution of cis and trans features, feature groups were randomly permuted and the AUROC loss was measured in leave‐out data. Feature groups were listed in Table S2 (Supporting Information). The AUROC loss of the *i*th feature group was calculated as:

(2) $$AUROC_{loss}=\left(AUROC-AUROC_{i}\right)/AUROC\times 100\%$$

where *AUROC* represents the AUROC value of prediction using entire feature set without permutation and *AUROC_i_* represents the AUROC value of prediction with *i*th feature group permuted.

### Analysis of the Effect of NOVA1 and NOVA2 on circRNA Biogenesis

A mouse brain dataset (GSE69711) which consists of three‐replicate samples for mouse cortex from embryonic 18.5‐day wild type, *Nova2*‐/‐, and *Nova1*‐/‐ mice was used to analyze the effect of Nova1 and Nova2 on circRNA biogenesis.^[^ ^42^ ^]^ The sequencing data (fastq file) was aligned to the mouse reference genome of version GRCm39 with bwa, and applied CIRIquant to identify and quantify circRNAs expressed in these samples. Differentially expressed circRNAs were identified by CIRI3 with a threshold of *pvalue* < 0.05. Binding peaks of *Nova1* and *Nova2* generated by HITS‐CLIP (bed files) were also downloaded from the same study. For Nova1 and Nova2, the average binding depth was estimated in the region from upstream 1000 bp to downstream 150 bp surrounding the upstream junction site, and from upstream 150 bp to downstream 1000 bp surrounding the downstream junction site. The average depth was calculated using a 10‐nt sliding window.

### DSC Inference with Poly(A) Selected Data

The same pipeline of bwa and CIRIquant as described above to identify and quantify circRNAs expressed in poly(A) RNA‐seq samples was used. For each sample comparison, only circRNAs detected in at least one poly(A) RNA‐seq sample were used for evaluation of DSC inference. The Δ*junction* *ratio* was used directly for inferring differentially spliced circRNA. If the circRNA was not detected, then the junction ratio was labeled as 0. The circRNA events with |*Δjunction ratio*| < 0.05 was labeled as “unchanged”, circRNA events with *Δjunction ratio* ≥ 0.05 was labeled as “higher in sample A” and circRNA events with *Δjunction ratio* ≤ −0.05 was labeled as “higher in sample B”.

### Inferring Regulated circRNAs in Cancer with TCGA Data

CIRI‐deepA was applied to TCGA datasets to detect consistently regulated circRNAs.^[^ ^43^ ^]^ Nine cancer datasets were used in this study: TCGA‐STAD, TCGA‐BRCA, TCGA‐COAD, TCGA‐KIRC, TCGA‐LIHC, TCGA‐LUAD, TCGA‐LUSC, TCGA‐PRAD and TCGA‐THCA. To avoid confounders introduced by different individuals, prediction was performed between samples collected from the same individual. We downloaded TPM gene expression data from TCGA repository. A circRNA was labeled as upregulated in a sample comparison if the prediction value corresponding to cancer sample was higher than 0.35, and was labeled as downregulated if the prediction value corresponding to normal sample is higher than 0.35. For each cancer type, circRNAs labeled as upregulated in more than 35% of the sample comparisons were defined as consistently upregulated circRNAs, and circRNAs labeled as downregulated in more than 35% of the sample comparisons were defined as consistently downregulated circRNAs of that cancer type.

### Inferring Regulated circRNAs in Glioma Dataset with Smart‐seq2 scRNA‐seq Data

A Smart‐seq2 dataset (GSE84465) to evaluate the applicability of CIRI‐deepA for differentially spliced circRNA inference using single cell RNA‐seq data was downloaded.^[^ ^44^ ^]^ In the dataset, a total of 3589 cells of different cell types (myeloid, oligodendrocytes, endothelial, neurons, astrocytes) were selected using cell type markers from human primary glioblastoma and periphery normal tissue samples.

The raw gene counts of all cells were downloaded from GEO repository. Seurat v4.3.0 to process the scRNA‐seq data was used.^[^ ^45^ ^]^ Cells with less than 10 genes detected and genes expressed in less than 200 cells were filtered out. The gene expression profile was normalized using “LogNormalize”, and 2000 highly variable genes were extracted and scaled for downstream analysis. Next, PCA using the previously determined variable genes was performed and the top 20 components were chose for UMAP dimension reduction and visualization. The cell type labels were assigned to the cell types given in the previous study.

For each cell, circRNAs expressed were identified and quantified using CIRIquant. For each cell types, total BSJ read counts and FSJ read counts in cells expressing the given circRNA were used to calculate junction ratios, and the junction ratios are next compared between tumor and normal groups. Due to low sequencing depth and data sparsity, circRNAs were only detected in limited cells, and only cells detected to express the given circRNA were included in differentially spliced circRNA analysis to avoid underestimation of the junction ratio. We employed DARTS BHT to infer the differentially spliced and unchanged circRNAs. CircRNAs with *p*(|*Δjuntion ratio*| > 0.05) > 0.9 and *MLE*(*junction ratio_tumor_* – *junction ratio_periphery_*) > 0 were labeled as “upregulated”, circRNAs with *p*(|*Δjuntion ratio*| > 0.05) > 0.9 and *MLE*(*junction ratio_tumor_* – *junction ratio_periphery_*) < 0 were labeled as “downregulated”, circRNAs with *p*(|*Δjuntion ratio*| > 0.05) < 0.1 were labeled as “unchanged”. In total, 67 circRNA events were inferred and labeled.

Then, CIRI‐deepA was applied to predict differentially spliced circRNAs between tumor and normal groups for each cell type. For each RBP, the mean expression value across cells was used to represent its expression in this cell type. Figure 4d showed the prediction value of the most abundant circRNAs that were detected to be expressed in at least 10 cells in both tumor and normal tissues.

### Inferring Cell Type Specific circRNAs

Another glioma scRNA‐seq dataset (GSE131928) was downloaded for evaluation.^[^ ^46^ ^]^ Cells in the sample were collected from IDH (isocitrate dehydrogenase) wild‐type glioblastomas and were processed through the 10X Chromium 3′ Single Cell Platform using the Chromium Single Cell 3′ Library.

The TPM gene expression matrix was filtered as described in last part, and then normalized as *TPM_norm_* = *ln*(*TPM*/100 + 1) for downstream analysis. The normalized data was integrated to the Smart‐seq2 glioma dataset to get circRNA expression. Features for integration was selected using the “SelectIntegrationFeatures” function, then two datasets were merged using the “FindIntegrationAnchors” and “IntegrateData” functions with default parameters provided by Seurat. The expression values were then scaled and the heterogeneity associated with platform was regressed out using the “ScaleData” function. PCA was performed on the integrated data, and the top 20 PCs were selected for defining clusters using the “FindNeighbors” and “FindClusters” functions in resolution of 0.3. As the cell composition of these two glioma datasets was not identical, to make sure that each cell type from two platforms have enough cells to define or predict cell type specific circRNA, clusters of which cells are less than 100 or cell proportion is less than 0.05 in at least one of the platforms were filtered out. The remaining clusters were then re‐clustered and visualized using UMAP. Cell type marker genes were used for classification of the remaining cell types, including *PTPRC* for myeloid and *GPR17*, *EDIL3* and *PLLP* for OPC. Neoplastic cells were split into two clusters. While both clusters have relatively high expression of *EGFR*, Neoplastic 1 cluster has relatively high expression of *C1R* and *ID3* and neoplastic 2 cluster has relatively high expression of *XRCC6BP1* and *STMN2*.

We defined cell type specific circRNAs according to the following criteria:

1. The circRNA were expressed in more than five cells of a cell type.
2. The mean junction ratio of the circRNA was higher than 0.05 in cells expressing the circRNA of the cell type.
3. P‐value of the one‐sided Wilcoxon rank sum test was less than 0.05 when comparing the junction ratios in the cell type against those in all other cells.

The prediction was performed between the given cell type and other clusters for each previously defined cell type specific circRNA. The circRNAs with the probability of higher junction ratio in the cluster higher than 0.35 were predicted to be cell type specific. The accuracy for each previously defined cell type specific circRNA is calculated as *Accuracy* = (*TP* + *TN*)/*N*, where *TP* represents the number of cell types predicted to have higher junction ratio in defined cell types, *TN* represents the number of cell types predicted to have lower junction ratio in other cell types and *N* represents the number of cell types. The random prediction was generated according to a uniform distribution with the interval from 0 to 1 for each circRNA and each cell type.

### Inferring Region Specific circRNAs in Spatial Transcriptomic Data

The filtered gene expression matrix (raw count) of a human developing heart dataset consisting of 19 samples from three development stages was downloaded from https://www.spatialresearch.org.^[^ ^47^ ^]^ The dataset was generated using the Spatial Transcriptomics v1.0 protocol. In a previous study, all sequenced spots were clustered into 10 clusters representing different anatomical regions as shown in Figure S9 (Supporting Information). Each section was split into four regions, including ventricle (cluster 0–3), atrium (cluster 4), outflow tract/large vessel (cluster 5) and other (cluster6‐9), to simplify the analysis.

Bulk transcriptomic data from RNAAtlas was used as reference to evaluate the predictions of CIRI‐deepA. Five bulk samples were used to extract region specific circRNAs: vena cava tissue (GSM4117975) representing outflow tract/large vessel regions, left atrium tissue and right atrium tissue (GSM4117981 and GSM4117987) representing atrium regions, and left ventricle tissue and right ventricle tissue (GSM411794 and GSM4117990) representing ventricle regions.

For each tissue, we defined a set of tissue specific circRNAs using the following criteria:

1. We calculated *τ* value of each circRNA across three tissue types with $\tau =\sum_{i=1}^{N}\left(1-x_{i}\right)/\left(N-1\right)$, where *x_i_* represents the junction ratio of circRNA normalized by the max junction ratio and *N* represents the number of tissues, three here. The *τ* value of tissue specific circRNAs should be higher than 0.5.
2. The junction ratio difference of tissue‐specific circRNA between the corresponding tissue and other tissues should be larger than 0.2.

Tangram was used to perform gene expression value imputation for each spot,^[^ ^48^ ^]^ using gene expression values from the corresponding scRNA‐seq data as reference. Top 100 genes of each cell type were used for mapping. The imputed RNA expression value of each spot was normalized with a scale factor of 300 000 and then used for prediction with CIRI‐deepA.

Then CIRI‐deepA was used to predict circRNAs with higher junction ratio for each anatomical region when compared to other regions. Fisher's exact test was used to test whether the predicted circRNAs are enriched in the region‐specific circRNAs. Random RBPs were generated by permutation over the entire gene expression profile and then used for prediction. The test with randomly selected circRNAs was also performed, the number of which was equal to the previously defined region‐specific circRNAs, denoted as “Random_label”.

### CircRNA Index for Generating Relative Junction Ratio Map

A circRNA index to indicate the relative junction ratio of a given circRNA in each region or spot was created. The index value was based on the prediction between the target region and the whole panel. Specifically, the index of the *i*th circRNA in *j*th region was calculated as follows:

(3) $$index_{i,j}=\left(1-p_{i,j}^{unchanged}\right)\times \left(p_{i,j}^{region}-p_{i,j}^{panel}\right)$$

where $p_{i,j}^{unchanged}$, $p_{i,j}^{region}$ and $p_{i,j}^{panel}$ represent three probabilities output by CIRI‐deepA when comparing the junction ratio of the *i*th circRNA in the *j*th region to the whole panel.

### Inferring Cell Type Proportion with Prediction Value Derived from CIRI‐deepA

A glioma dataset generated with 10X Visium in study of Ravi et al. was downloaded.^[^ ^49^ ^]^ The dataset was used to evaluate the applicability of prediction values derived from CIRI‐deepA in predicting cell type proportion. We used CARD to infer the cell type proportion of each spot using the Smart‐seq2 scRNA‐seq glioma dataset mentioned above as reference,^[^ ^26^ ^]^ with parameters “minCountGene = 100, minCountSpot = 5”. Then CIRI‐deepA was applied to generate three probabilities comparing each spot to the whole panel. 18 circRNAs derived from the Smart‐seq2 dataset with high cell type specificity (*τ* value > 0.75) were selected for prediction.^[^ ^50^ ^]^ Regression models for three major cell types (myeloid, neoplastic1 and neoplastic2) using the prediction values corresponding to the spot and the whole panel as predictor was developed. We use “glmnet” package in R to build the three LASSO models with an internal 10‐fold cross‐validation,^[^ ^51^ ^]^ with the lambda that minimizes the cross‐validation error.

## Conflict of Interest

The authors declare no conflict of interest.

## Author Contributions

Z.Z. and J.Z. contributed equally to this work. F.Z and Y.G. conceived the project. Z.Z. Z.P. and Y.G. designed the method. Z.Z. and J.Z. implemented the model. Z.Z., J.Z. and X.Z. conducted the analysis. Z.Z., F.Z and Y.G. wrote the manuscript with contributions from all authors.

## Supporting information

Supporting Information

Supporting Information

Supporting Information

Supporting Information

## Data Availability

The data that support the findings of this study are available in the supplementary material of this article.
