# Supplementary material for: CIRI‐Deep Enables Single‐Cell and Spatial Transcriptomic Analysis of Circular RNAs with Deep Learning
Source: Adv Sci (Weinh). 2024 Feb 2;11(14):2308115. doi: 10.1002/advs.202308115 (PMC11005702; doi:10.1002/advs.202308115)
Supplement: Supplementary file 1 — Supporting Information [file ADVS-11-2308115-s004.pdf]

ADVANCED SCIENCE
Open Access

Supporting Information

for *Adv. Sci.*, DOI 10.1002/advs.202308115

CIRI-Deep Enables Single-Cell and Spatial Transcriptomic Analysis of Circular RNAs with Deep Learning

Zihan Zhou, Jinyang Zhang, Xin Zheng, Zhicheng Pan, Fangqing Zhao and Yuan Gao**

Supporting Information

CIRI-deep Enables Single-cell and Spatial Transcriptomic Analysis of Circular RNAs with Deep Learning

Zihan Zhou[1,3,5], Jinyang Zhang[2,3,5], Xin Zheng[1,3], Zhicheng Pan[4], Fangqing Zhao[2,3], Yuan Gao[1,3]**

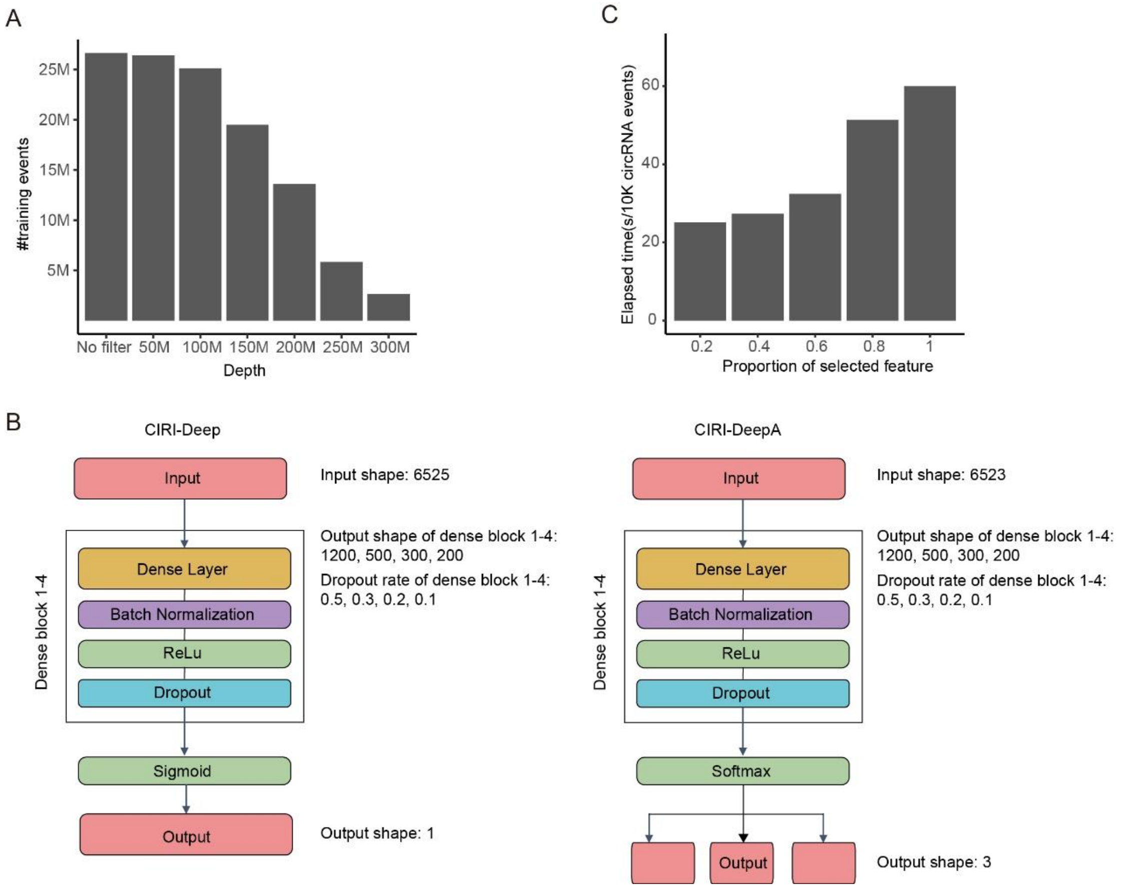

Figure S1. Model structure and training process

A. Number of training events at different depth thresholds.

B. Model structures of CIRI-deep and CIRI-deepA. Hyperparameters are shown on the right.

C. Running time of CIRI-deepA model with different size of input features. The x-axis represents the proportion of selected features in the whole feature set. The same proportion of neurons were kept in hidden layers. The y-axis represents elapsed time used to predict 10K circRNA events.

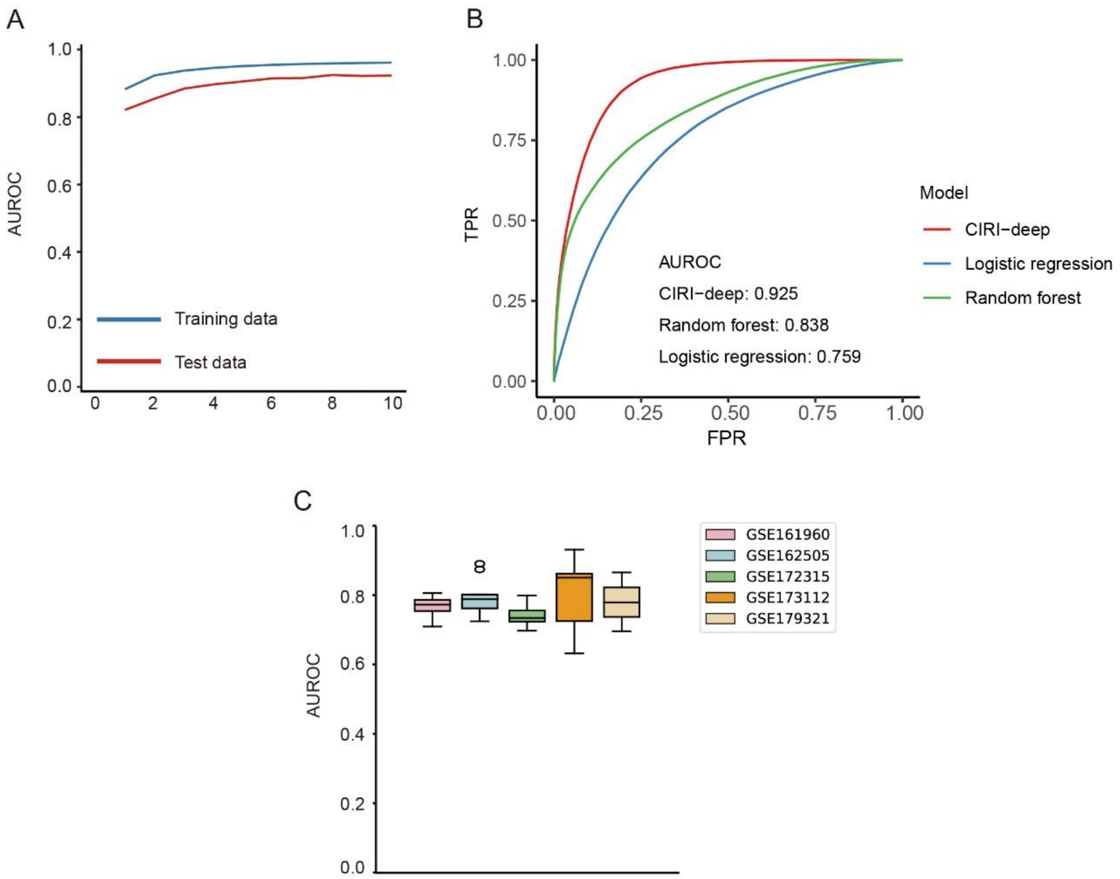

Figure S2. Performance of CIRIdeep

A. AUROC of CIRI-deep on training data and test data during training process.

B. AUROC curve of three models (CIRI-deep, Logistic regression, Random forest) on test data.

C. AUROC of CIRI-deep on 5 public datasets.

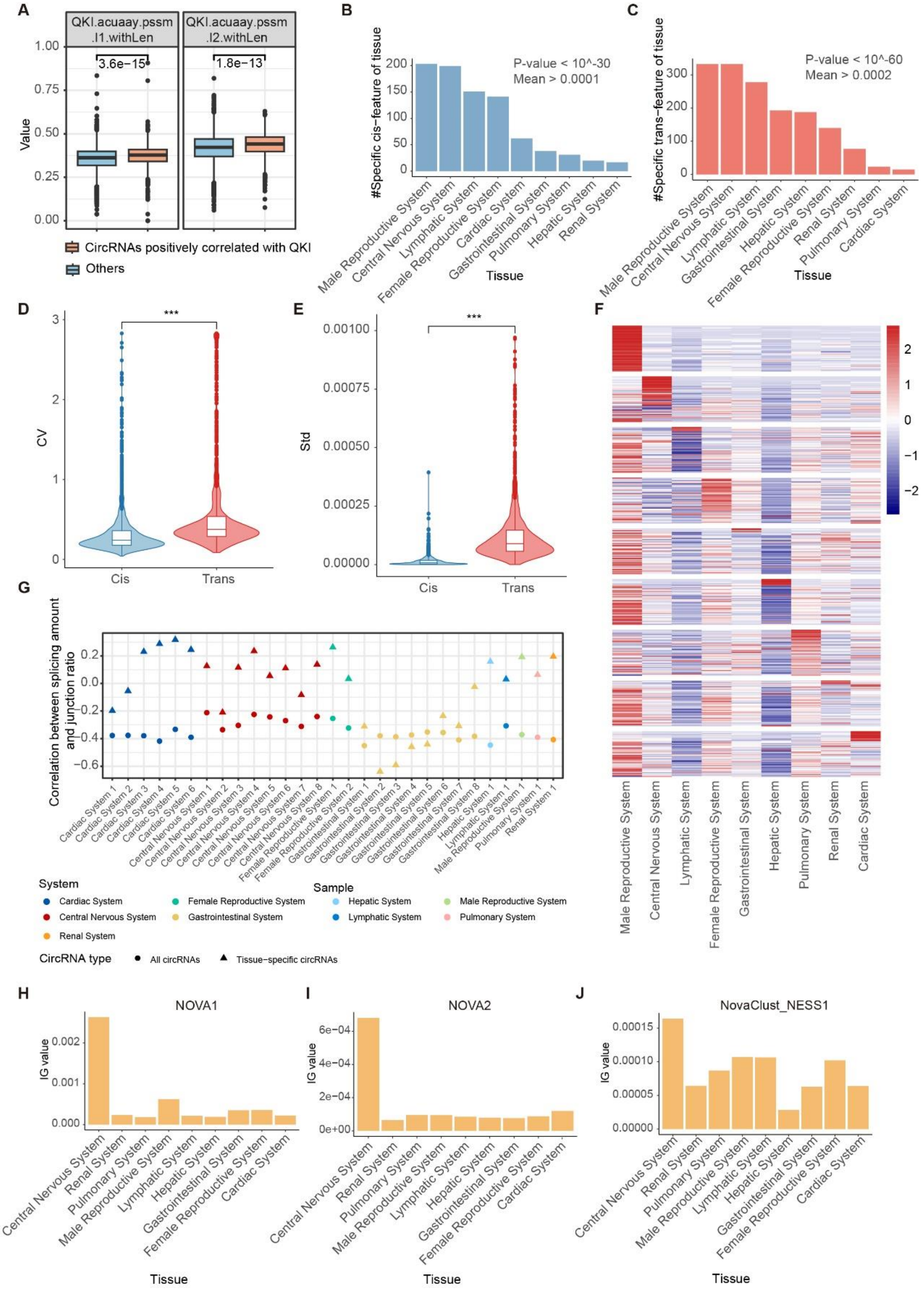

Figure S3. Model interpretation revealed tissue-specific cis and trans features

A. QKI binding motif density in flanking introns of circRNAs positively correlated with QKI expression level and other circRNAs. Y-axis represents the value of the QKI binding motif density in flanking introns. Orange and blue boxes represent circRNAs correlated with QKI expression level and other circRNAs. Left panel and right panels represent QKI binding motif density in upstream and downstream introns, respectively.

B-C. Number of tissue-specific cis and trans features across 9 tissues. Thresholds used for filtering were shown on upper-right.

D-E. Coefficient of variation (CV) and standard deviation of cis and trans features across 9 tissues.

F. Expression level of RBPs with top IG values in nine tissues.

G. Spearman correlation between splicing amount (SRPM) and junction ratio of all expressed circRNAs and tissue-specific circRNAs across nine tissues. X-axis represents different samples of nine tissues. Y-axis represents spearman correlation (rho). Expressed circRNAs of each sample were labeled as circle and tissue-specific circRNAs of each tissue were labeled as triangle.

H-J. IG value of *NOVA1*, *NOVA2* (trans feature) and NovaClust_NESS1 (cis feature) across 9 tissues.

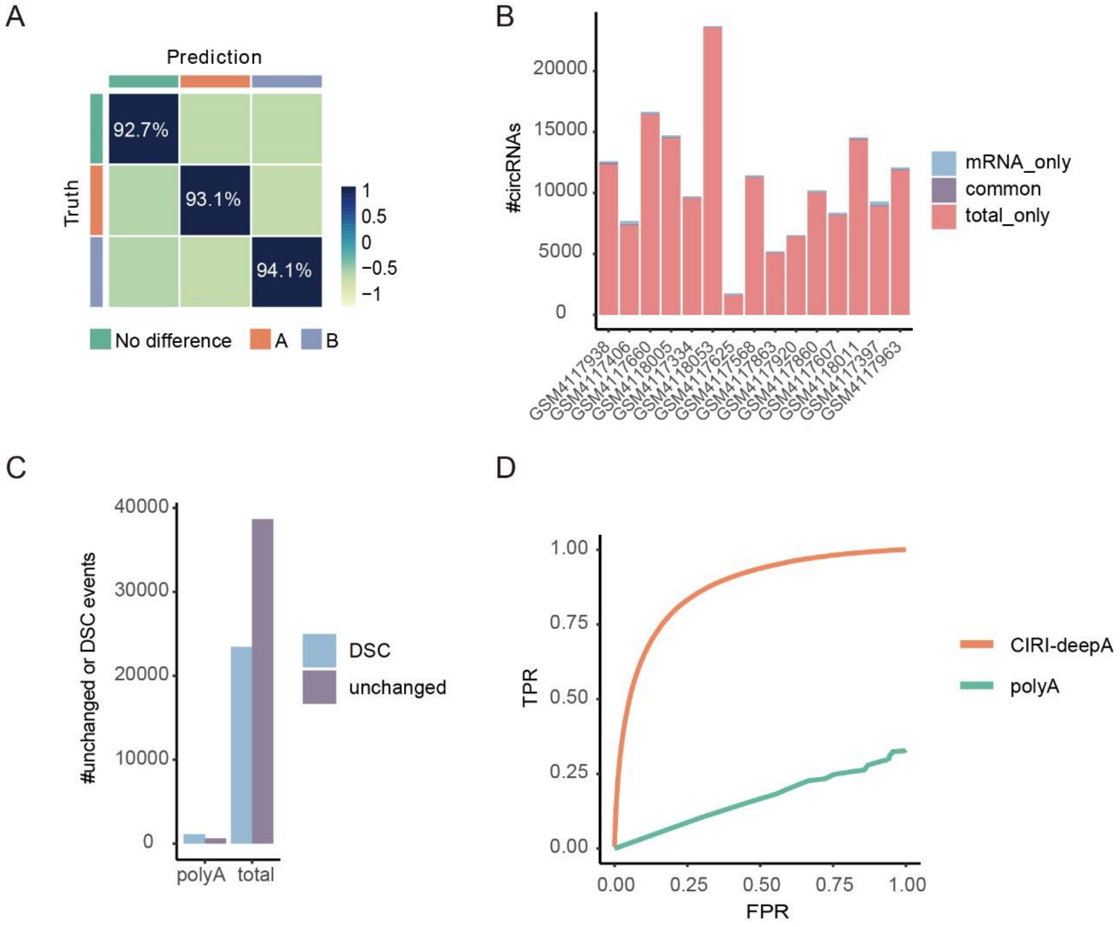

Figure S4. CIRI-deepA were able to predict DSC from bulk and single cell poly(A) RNA-seq data

A. Performance of model trained on poly(A)-enriched RBP expression data. The heatmap showed accuracy on test data of 3 classes: no difference, higher junction ratio in sample A and higher junction ratio in sample B, scaled by row. Precision of each class was shown on the heatmap.

B. Number of circRNAs detected in total RNA datasets and corresponding mRNA datasets.

C. Number of unchanged or DSC events detected among poly(A)-enriched transcriptome sample pairs or total RNA transcriptome sample pairs.

D. ROC curve of DSC discrimination using CIRI-deepA (orange line) or with junction ratio difference calculated from poly(A)-enriched data (green line).

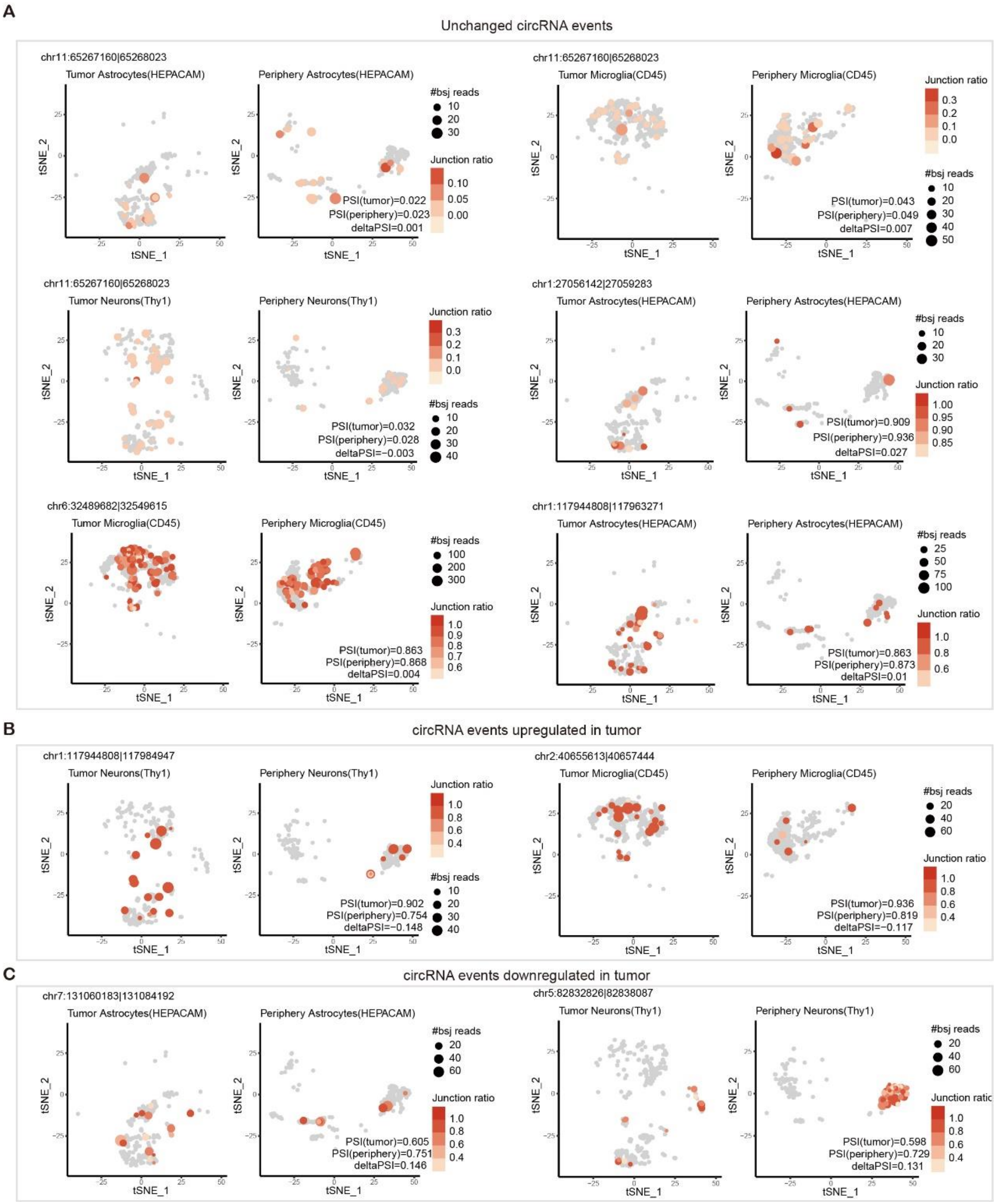

Figure S5. 10 most abundant unchanged or DSC events between glioma tissue and periphery tissue in Smart-seq2 datasets.

Cells with circRNA detected labeled with size and color indicating number of back-splicing reads and junction ratio of the circRNA in the cell. Junction ratio of tumor and periphery tissue and difference of junction ratio value were shown on the plot. Upper block, middle block and lower block are circRNAs unchanged between tissue, with higher junction ratio in tumor tissue and with higher junction ratio in periphery tissue, respectively.

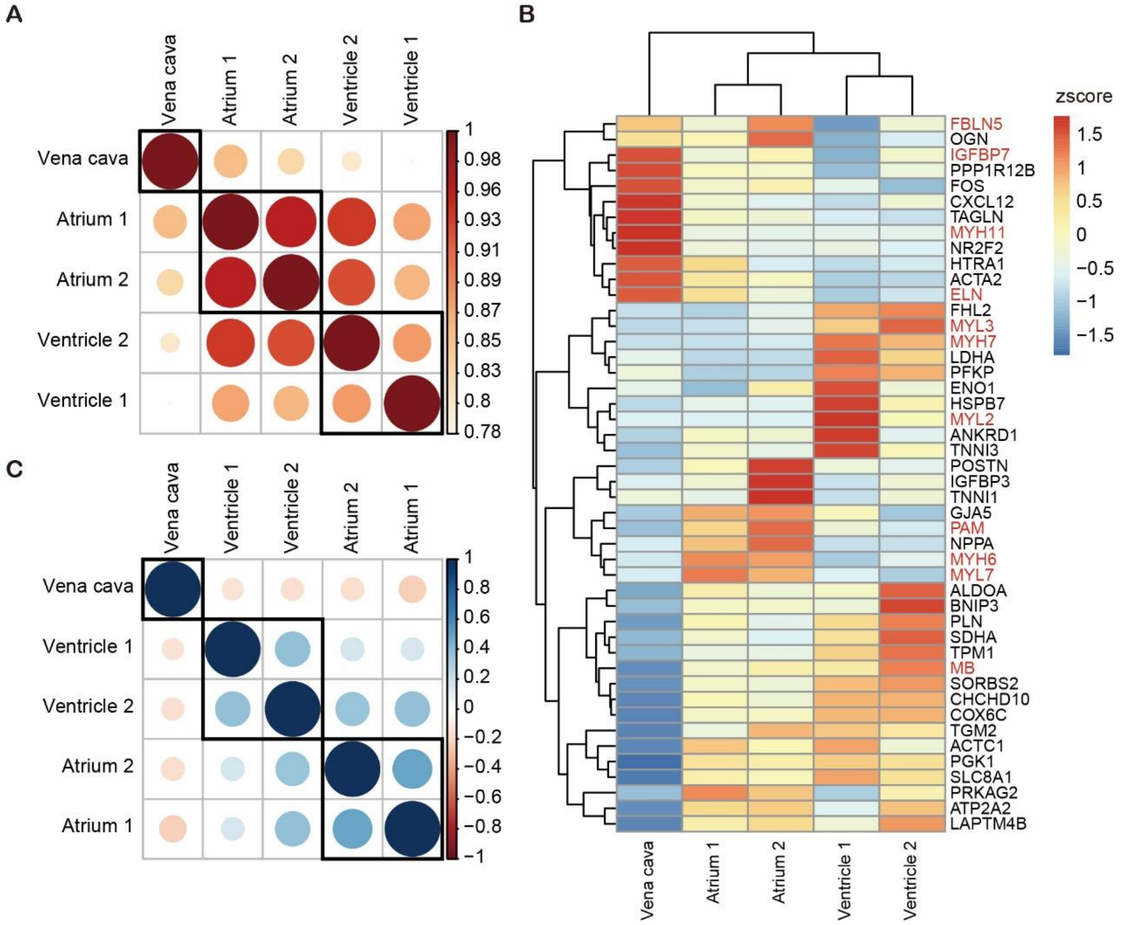

Figure S6. Bulk tissue from RNAAtlas corresponded to three sub regions

A. Gene expression profile correlation (spearman) between each pair of bulk tissues.

B. Expression profile of marker gene of ventricle, atrium and vena cava. Highlighted genes are top differentially expressed gene identified in previous study.

C. Region specific circRNAs junction ratio correlation (pearson) between each pair of bulk tissues.

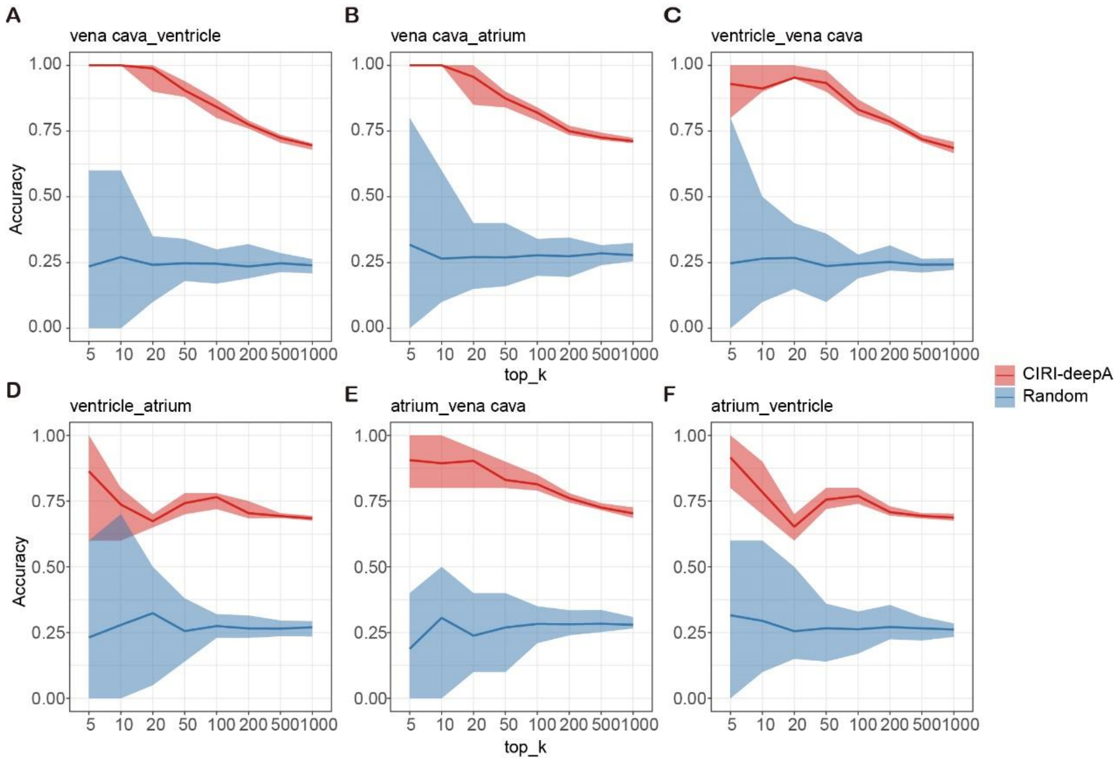

Figure S7. Top k accuracy of DSCs predicted by CIRI-deepA

A-F. Top k accuracy of DSCs between outflow tract / atrium / ventricle region predicted by CIRI-deepA. Upper and lower bound of colored region indicate the max and minimum value of prediction result of 19 tissue sections; line indicates mean value. Random prediction was carried by randomly chosen *k* circRNAs as predicted DSCs from the whole circRNA set (23459).

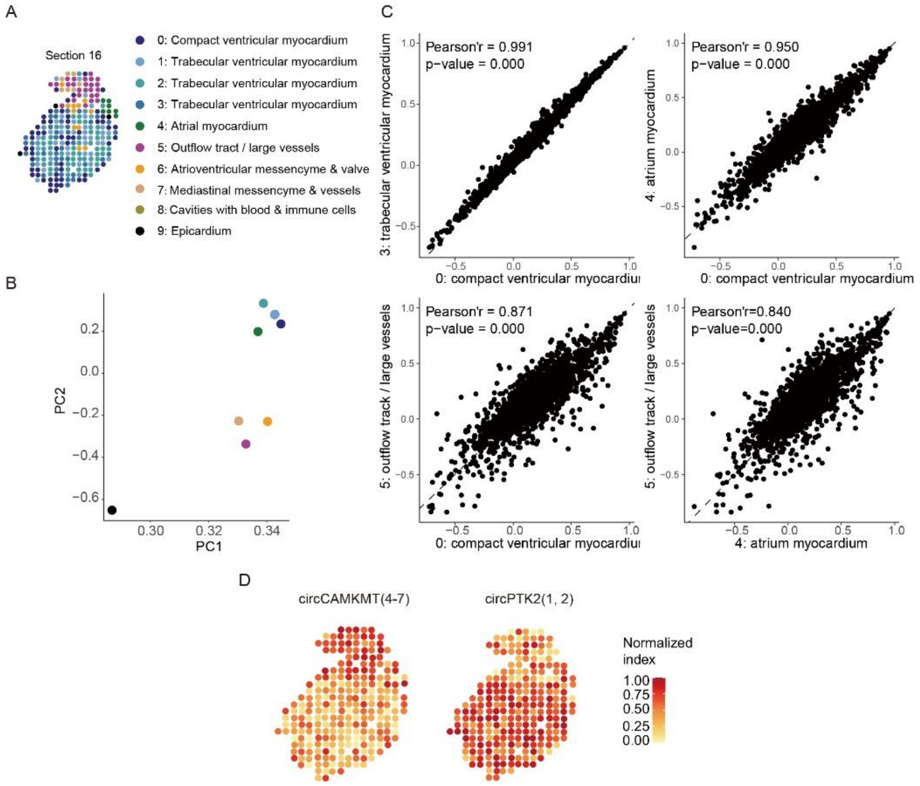

Figure S8. CircRNA region and spot index

A. Spatial positions of 9 anatomical regions (label of regions are annotated based on clustering of 3,115 spots of 19 tissue sections of human embryonic hearts) in tissue section 16. There is no spot assigned to cluster 8 in section 16.

B. Dimensionality reduction of circRNA region index profile (consisting of 23,459 circRNAs) in 9 regions of section 16.

C. Pearson correlation between index of circRNAs in regions.

D. CircRNA spot index plot of circCAMKMT(4-7) and circPTK2(1, 2).

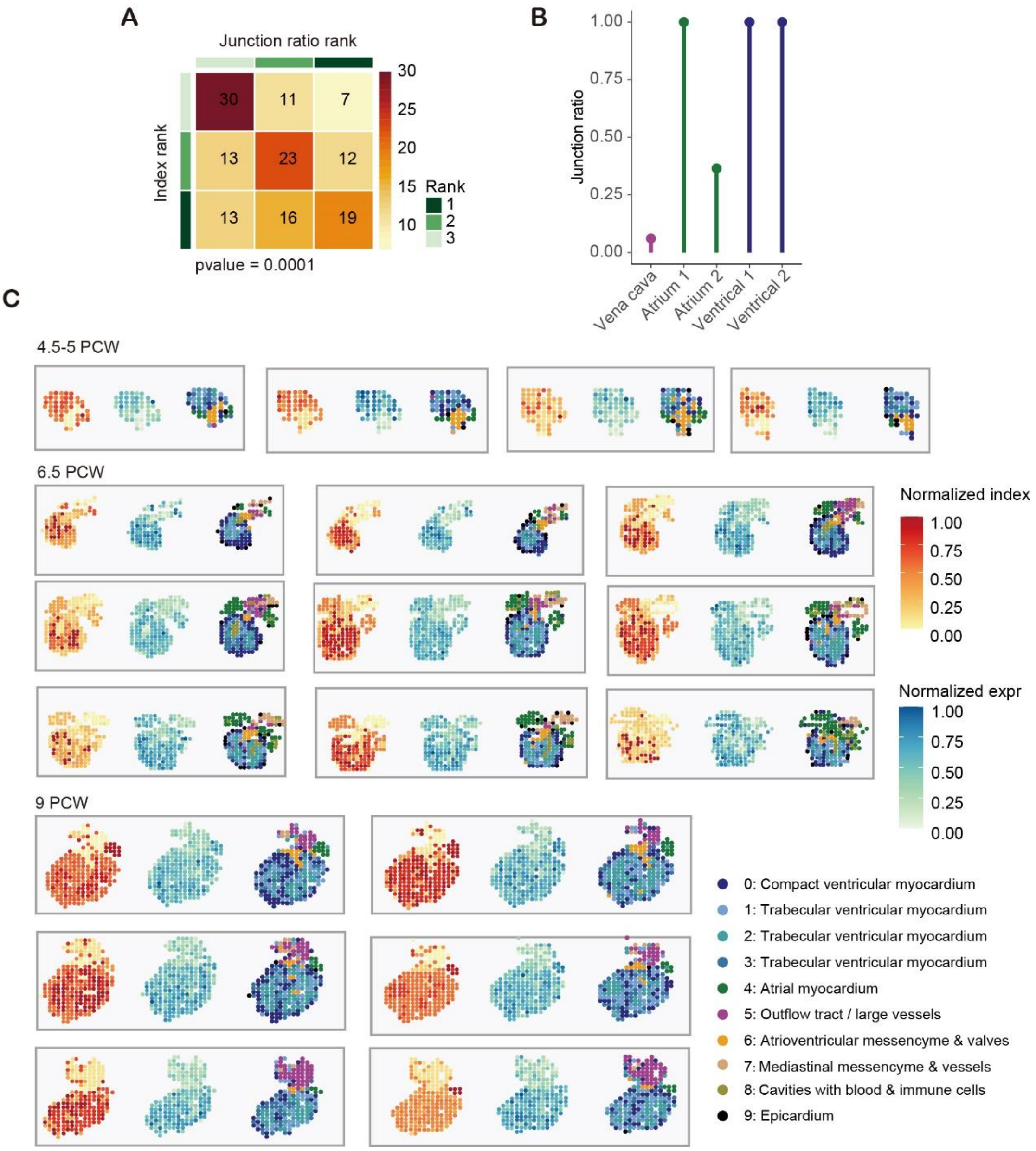

Figure S9. Region circRNA index of human developing heart

A. Rank correlation of circRNA index among three sub regions (outflow tract / ventricle / atrium) and junction ratio among three types of bulk tissues (vena cava / ventricle / atrium). Pvalue was labeled on the bottom of heatmap.

B. Junction ratio of circQKI(2-4) in 5 bulk tissues.

C. circQKI(2-4) relative junction ratio plot (left), QKI relative expression (middle) and histological regions (right) of 19 human developing heart tissue sections across 3 development stage.

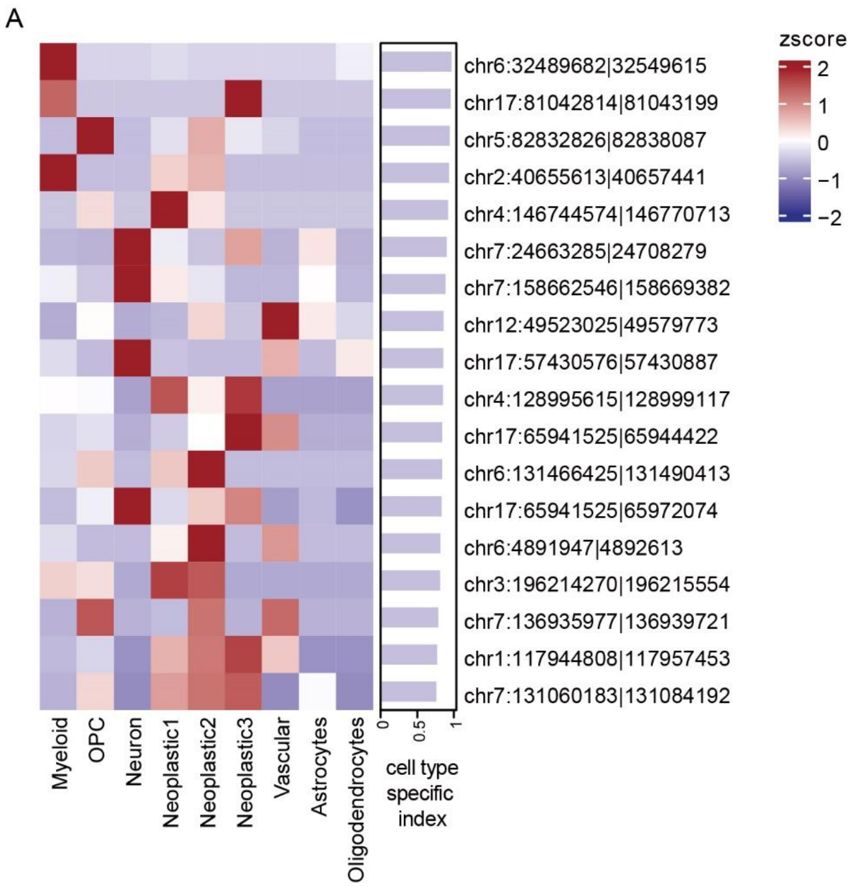

Figure S10. 18 circRNAs used for cell type proportion prediction

The junction ratio (scale by row) of 18 circRNAs in 9 cell types were shown in the heatmap. The bar indicated the cell type specific index of these circRNAs (all of them are higher than 0.75).
